# Stress-Driven Discovery of New Angucycline-Type Antibiotics from a Marine *Streptomyces pratensis* NA-ZhouS1

**DOI:** 10.3390/md16090331

**Published:** 2018-09-12

**Authors:** Najeeb Akhter, Yaqin Liu, Bibi Nazia Auckloo, Yutong Shi, Kuiwu Wang, Juanjuan Chen, Xiaodan Wu, Bin Wu

**Affiliations:** 1 Ocean College, Zhejiang University, Hangzhou 310058, China; na_memon@yahoo.com (N.A.); naz22ia@hotmail.com (B.N.A.); 11434028@zju.edu.cn (Y.S.); 2 Department of Chemistry, Zhejiang University, Hangzhou 301000, China; yaqin86@zju.edu.cn; 3 Department of Chemistry, Zhejiang Gongshang University, Hangzhou 310012, China; wkwnpc@zjgsu.edu.cn; 4 Key Laboratory of Applied Marine Biotechnology, Ningbo University, Chinese Ministry of Education, Ningbo 315211, China; chenjuanjuan@nbu.edu.cn; 5 Centre of Analysis and Measurement, Zhejiang University, Hangzhou 310058, China; wxd_zju@163.com

**Keywords:** marine microorganisms, *Streptomyces pratensis*, polyketide antibiotics, metal stress technique, antimicrobial activity

## Abstract

Natural products from marine actinomycetes remain an important resource for drug discovery, many of which are produced by the genus, Streptomyces. However, in standard laboratory conditions, specific gene clusters in microbes have long been considered silent or covert. Thus, various stress techniques activated latent gene clusters leading to isolation of potential metabolites. This study focused on the analysis of two new angucycline antibiotics isolated from the culture filtrate of a marine *Streptomyces pratensis* strain NA-ZhouS1, named, stremycin A (**1**) and B (**2**) which were further determined based on spectroscopic techniques such as high resolution time of flight mass spectrometry (HR-TOF-MS), 1D, and 2D nuclear magnetic resonance (NMR) experiments. In addition, four other known compounds, namely, 2-[2-(3,5-dimethyl-2-oxo-cyclohexyl)-6-oxo-tetrahydro-pyran-4yl]-acetamide (**3**), cyclo[l-(4-hydroxyprolinyl)-l-leucine] (**4**), 2-methyl-3*H*-quinazoline-4-one (**5**), and menthane derivative, 3-(hydroxymethyl)-6-isopropyl-10,12-dioxatricyclo[7.2.1.0]dodec-4-en-8-one (**6**) were obtained and elucidated by means of 1D NMR spectrometry. Herein, we describe the “Metal Stress Technique” applied in the discovery of angucyclines, a distinctive class of antibiotics that are commonly encoded in microbiomes but have never been reported in “Metal Stress” based discovery efforts. Novel antibiotics **1** and **2** exhibited antimicrobial activities against *Pseudomonas aeruginosa*, methicillin resistant *Staphylococcus aureus* (MRSA), *Klebsiella pneumonia*, and *Escherichia coli* with equal minimum inhibitory concentration (MIC) values of 16 µg/mL, while these antibiotics showed inhibition against *Bacillus subtilis* at MIC value of approximately 8–16 µg/mL, respectively. As a result, the outcome of this investigation revealed that metal stress is an effective technique in unlocking the biosynthetic potential and resulting production of novel antibiotics.

## 1. Introduction

In the microbial world, secondary metabolites may act as natural antibiotics, enzyme inhibitors, pigments, and toxins for microbial protection or behave as signaling agents depending on their concentrations [1,2,3,4]. In spite of the fact that above half of all medications are based on terrestrial natural products platforms, the marine habitat that comprises 71% of the Earth’s surface may provide an exceptional possibility to explore novel therapeutics because of its unusual chemical diversity and growth conditions [5]. The exploration of new natural products from marine resources led to the isolation of about 15,000 novel secondary metabolites during the period of 2001–2015 [6]. Marine microorganisms usually thrive under distinctive conditions like temperature, pressure, dissolved oxygen, and nutrient availability, leading to the production of structurally and biologically interesting compounds. As such, marine actinomycetes have been revealed as an incredible source of novel secondary metabolites with various biological activities [7,8,9,10,11,12,13,14,15]. More specifically, marine *Streptomyces* derived compounds have demonstrated their potency to exhibit cytotoxic, anticancer, antifungal, and antimicrobial effects such as warkmycin, 12-deoxo-12-hydroxy-8-*O*-methyltetrangomycin, marizomib, and salinosporamide A [16,17,18,19]. Abiotic strategies such as chemical stress (heavy metal), biotic stress (co-cultivation), and changes in fermentation conditions (light, pH, temperature, and various media) are long known to induce notable changes or function to unlock cryptic biosynthetic gene clusters in the microbial metabolome [4,20]. Standard laboratory culture conditions have proven to hinder activation of specific gene clusters which, in turn, hamper the generation of secondary metabolites. Previous work which we have conducted demonstrates the successful utility of the “metal stress” strategy for activating silent gene clusters and subsequent isolation of unique natural products which exhibit potent antimicrobial properties [5,21,22,23].

The angucycline group of antibiotics belongs to a specific group of polycyclic aromatic polyketides derived from naturally occurring quinone saccharide antibiotics, which exhibit mainly anticancer and antimicrobial activities [24,25]. This type of antibiotic was first discovered as a tetrangomycin isolated from *Streptomyces rimosus* in 1965 and was shown to have a C–C bond connectivity with C-9 linked sugar moieties [17,26]. A large number of angucyclines are produced as *C*-glycoside antibiotics, and displaythis element as one of the most distinctive and typical structural characteristics. It is also known that theseantibiotics are produced by actinomycetes with Streptomyces as the major producer [27].

**Figure 1:**
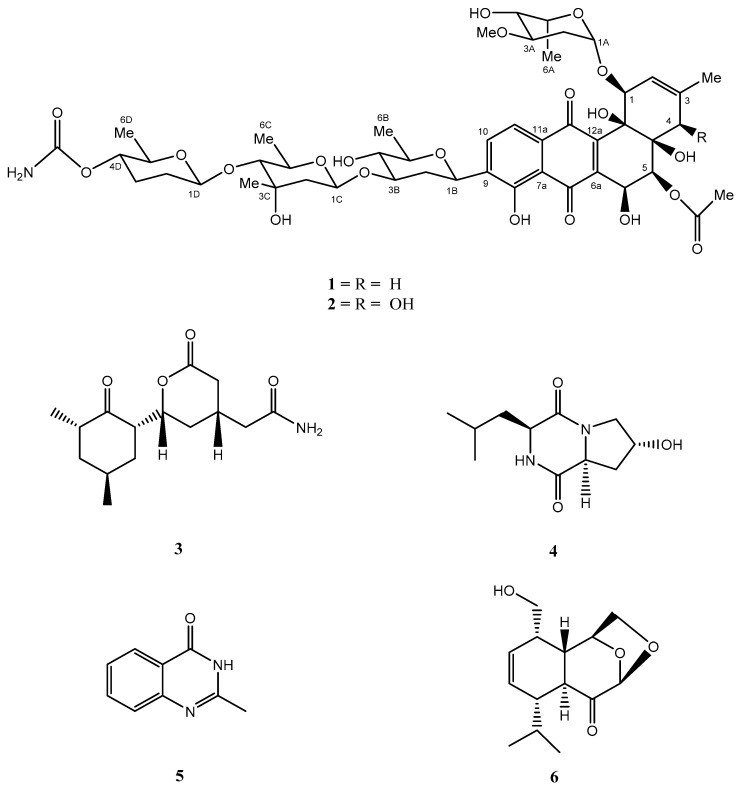
Chemical structures of stress metabolites **1**–**6**.

In order to discover new secondary metabolites and extend the use of the “Metal Stress” strategy that stimulates the cryptic gene cluster of marine microorganisms, different metal ions were applied to the marine Streptomyces strain NA-ZhouS1. Under one of these implemented conditions, referred to herein as heavy metal nickel (100 µM) followed by their antibacterial capacities together with a comparison of extract in high performance liquid chromatography (HPLC) profile, grasped our consideration, and facilitated interaction with new compounds stremycin A (**1**) and B (**2**). The results in the comparison revealed that the addition of metal induction would streamline natural product development efforts. Further, this study deals with the isolation, structure elucidation and bioactivities of two new aromatic polyketides **1** and **2**, in addition to known compounds **3**–**6**. The structures are shown in Figure 1.

## 2. Results

**Figure 2:**
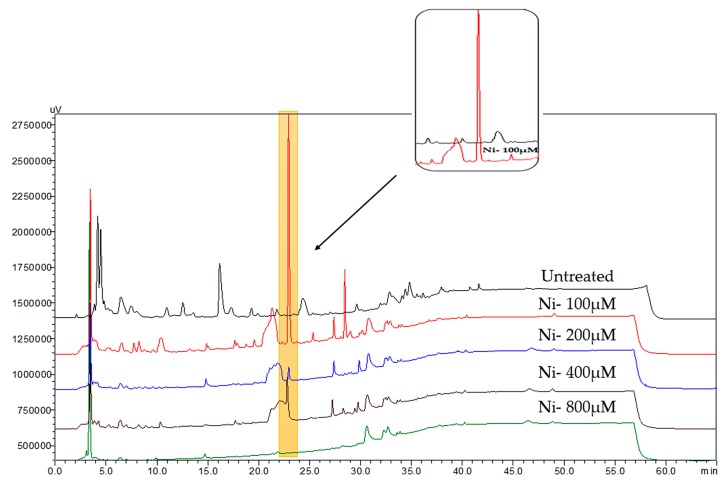
HPLC analysis metabolic profile of NA-ZhouS1 under nickel ion stress condition.

*Streptomyces pratensis* strain NA-ZhouS1 was isolated from marine sediment in the waters along the Zhoushan Coast in East China. Throughout this study, the strain was treated with the abiotic stress reagents, such as NiCl_2_·6H_2_O; CoCl_2_·6H_2_O; ZnSO_4_·7H_2_O; CrCl_3_·6H_2_O; MnCl_2_·6H_2_O at concentrations of 100, 200, 400, 800 µM, respectively. As a consequence, based on HPLC guided profile, 100 µM nickel ion (NiCl_2_·6H_2_O) was chosen as the best elicitor of stress in the *S. pratensis* strain toward the production of antibiotics in comparison to conditions used for normal growth of the strain (Figure 2). The 30 L of nickel treated culture broth was extracted with ethyl acetate (EtOAc) and subjected to reverse phase column using C18 silica gel, Sephadex LH-20, followed by further purification with preparative HPLC (flow rate 10 mL/min, ultraviolet (UV) detector 210 nm), which successfully led to the isolation of two new aromatic polyketides, namely, stremycin A (**1**) and B (**2**) together with a known compound **3**. Moreover, the other culture extract of the same strain, which was induced by zinc ion to a concentration of 100 µM, subjected to analytical HPLC (flow rate 0.8 mL/min, eluted mode 0~30 min 20%~100% (H_2_O/MeOH), 30–50 min 100% MeOH, UV detector 210 nm) led to the isolation of three known compounds **4**–**6**.

### Structural Elucidation of Novel Compounds

Stremycin A (**1**) was detected and isolated as a yellow powder, giving the molecular formula of C_48_H_65_NO_21_ according to the HR-TOF-MS analysis in positive ion mode at *m/z* 1014.3937 for [M + Na]^+^ (Calcd. 1014.3941) and in negative ion mode at *m/z* 990.3964 for [M − H]^−^ (Calcd. 990.3976), (Supplementary Material (SM), Figures S26 and S27). Carefully analysis of ^1^H and ^13^C NMR spectroscopic data exhibited features characteristic of tetracyclic benz[*α*]anthracene core, with the 1-position *O*-glycosylated and 9-position *C*-glycosylated, which were characterized by resonances corresponding to twenty methines, fourteen quaternary carbons, six methylene, and nine methyl groups (Table 1). These signals were comparable to those of warkmycin and P371A1 [16,28]. Although the structure of **1** was analogous to warkmycin whilea difference was observed in the substituent pattern, i.e., the presence of a carbamoyl group -CONH_2_in the region of sugar D instead of sugar A ofwarkmycin [16]. This was further confirmed following observation of heteronuclear multiple bond correlation (HMBC) cross peaks from H-4D to a carbamoyl carbon at (*δ*_C_ 159.6). Another difference wasnoted at C-4 (*δ*_C_ 36.3) position in the aglycone of **1**, where the methylene protons were seen at *δ*_H_ [1.99, 2.36 (d, *J* = 17.6)] instead of an oxygenated methine [*δ*_H_ 5.36, *δ*_C_ 68.1 (CH-4)] in warkmycin.Hence, the entire assignment of all the ^1^H and ^13^C NMR data of **1** was finally performed by the correlative analysis of its ^1^H-^1^H correlated spectroscopy (COSY), heteronuclear single quantum correlation (HSQC), HMBC, and nuclear overhauser effect spectroscopy (NOESY) experiments (SM, Figures S4–S6 and S8). The ^1^H NMR spectrum displayed a set of *ortho*-coupled aromatic proton signals appeared at *δ*_H_ 7.87, 7.64 (d, *J* = 7.9 Hz, H-10/11), two oxygenated methine protons at *δ*_H_ 5.84, 4.92 (d, *J* = 6.8 Hz, H-5/6), and an olefinic proton signal at *δ*_H_ 5.63 (1H, s, H-2), which were associated with the carbons resonated at *δ*_C_ 134.2 (C-10), 120.1 (C-11), 76.2 (C-5), 69.9 (C-6), and 76.2 (C-2), as seen via the HSQC spectrum results. These *ortho*-coupled protons (H-10/H-11 and H-5/H-6), showed diagnostic COSY contacts with typical ^1^H-^1^H coupling constants, which was extended by HMBC correlations from H-10 to C-7a, C-8, and C-11a; H-11 to C-7a, C-9, C-10, and C-12; H-5 to C-6; H-6 to C-5, C-6a, and C-12a to establish the connectivity of rings. The fusing pattern of another ring was deduced by observing the HMBC correlations from H_2_-4 to C-4a, C-5, and from H-1 to C-2, C-4a, and C-12b to complete the assignment of aglycone skeleton. In addition, a typical quinone analogs system was identified from the significant carbonyl chemical shifts, which were visible at *δ*_C_ 187.24 (C-12) and *δ*_C_ 190.52 (C-7) in the ^13^C NMR spectrum. A substituted singlet methyl resonance noticeable at (*δ*_H_ 1.68, *δ*_C_ 23.58) was confirmed at C-3 *δ*_C_ 136.61 by HMBC correlations of *δ*_H_ 1.68 to C-3, C-4, while an acetyl group resonated at *δ*_C_ 172.39 (5-COMe) was assigned to an oxygenated methine at [*δ*_H_ 5.84, *δ*_C_ 76.24 (CH-5)] by cross-peak correlations observed in the HMBC spectrum from H-5 to a quaternary carbon *δ*_C_ 172.39. As a consequence, a detailed analysis of two-dimensional (2D) nuclear magnetic resonance spectroscopy data was performed as compared to previously published literature. This revealed the *cis* arrangement with a strong correlation in between H-5 and H-6. Since H-4 showed a diagnostic NOESY cross peak with H-5, it indicated that the acetyl group was in an *α*-configuration. Since H-5 was *α*-oriented, no NOESY connection would be present between H-5 and H-4 owing to the bulky OAc group which stayed as equatorial, pushing H-5 away from both H-4. The coupling constant *J*_5–6_ = 6.8 Hz revealed the hydroxyl group at C-6 to be *α*-oriented.

In the 1D (^1^H, ^13^C) NMR spectrum, three acetal carbon resonances observed at *δ*_C_ 99.31 (C-1C), *δ*_C_ 100.64 (C-1A) and *δ*_C_ 104.57 (C-1D), as well as four doublet methyl proton resonances appeared at *δ*_H_ 1.18 (3H, d, *J* = 6.6 Hz, H-6A), 1.38 (3H, d, *J* = 6.1 Hz, 6B), 1.31 (3H, d, *J* = 6.1 Hz, H-6C), and 1.22 (3H, d, *J* = 6.2 Hz, H-6D) revealed the existence of four deoxy sugars, three of which *O*-linked and one needed to be *C*-glycosidically linked to the aglycone of **1**.

**Figure 3:**
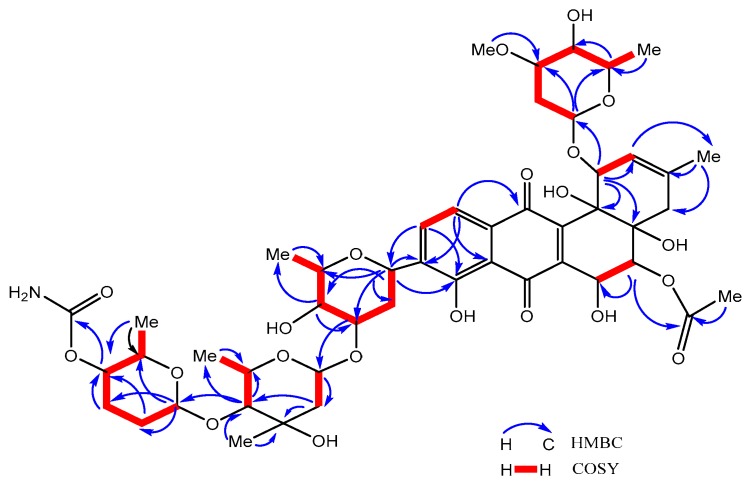
The key ^1^H-^1^H COSY, HMBC correlations of stremycin A (**1**).

A thorough analysis of the 2D NMR experiment was carried out to clarify the connection of four sugar units (A–D) attached to aglycone as shown in Figure 3. As such, in the substituent of sugar A, a small coupling constant (*J* = 4.0 Hz) of an anomeric proton resonated at *δ*_H_ 4.60 (H-1A) proved that this unit was *α*-*O*-glycosidically linked to angucycline core. Further, the observed ^3^*J*_C-H_ long-range correlations from H-1A to C-1 (*δ*_C_ 82.1) and H-1 to C-1A (*δ*_C_ 100.6) in the HMBC spectrum confirmed the connection of C-1-*O*-C-1A between the aglycone and oleandrose. The NOESY cross peaks of H-1 and H-1A revealed an axial orientation of H-1. Similarly the ^1^H-^1^H COSY correlations of H-1A/H-2A, H-3A/H-4A, H-5A/H-6A, and the HMBC correlations of H-1A to C-3A, C-5A revealed the presence of a six-membered deoxy sugar. Moreover, the singlet methoxy group resonated at (*δ*_H_ 3.24, *δ*_C_ 57.50) was confirmed at CH-3A by HMBC ^3^*J*_C-H_ long-range cross peaks. Comparison of our conclusions with those found in the literature that the sugar A is a known unit, namely, *α*-*O*-5-epi-oleandrose [16]. 

As such, the significant HMBC long-range correlations from the anomeric methine proton (CH-1B) resonated at (*δ*_H_4.89, *δ*_C_ 72.43) to C-8, C-9, and C-10 inferred the presence of *C*-glycosidic bond (C9-C1B) between the aglycone and olivose sugar moiety. The resonance of H-1B showed an overlapped peak in the ^1^H NMR spectrum, thus it was not possible to determine the exact coupling constant. Correspondingly, the coupling constant (*J* = 8.9 Hz) of a methine proton resonated at *δ*_H_ 3.11 (H-4B) revealed that sugar B assumes the acetal carbon (C-1) conformation in which all protons were axially oriented excluding the H-5B and H-6B. The hydroxyl group at CH-5B (*δ*_H_ 3.46, *δ*_C_ 77.7) and the methyl group of H-6B considered being equatorial when compared to those of warkamycin [16]. Further analysis was observed by ^1^H-^1^H COSY correlations in between H-1B/H-2B, H-2B/H-3B, H-3B/H-4B, H-4B/H-5B, and H-5B/Me-6B, followed by the HMBC correlations of H-1B to C-2, C-3, C4, and C-5 confirmed the presence of the sugar olivose. Hence, the combined results with comparison of published literature led to the identification of sugar B as *β*-*C*-olivose linked to C-9 on the angucycline core.

Similarly, the substituent of sugar C displayed large coupling constants *J*_H-1C_ = 9.9, 1.8 Hz and *J*_H-4C_ = 9.6 Hz resonated at *δ*_H_ 4.78 and at *δ*_H_ 3.19, signifying this unit as *β*-glycosidically bonded to sugars by also revealing an axial orientation. In addition, the connectivity of sugar B and C as *O*-glycosidic linkage C-3B-*O*-C-1C was deduced by HMBC long-range correlationsof H-3B to C-1C (*δ*_C_ 99.31). Moreover, the ^1^H-^1^H COSY correlations of H-1C/H-2C, H-4C/H-5C, H-5C/H-6C, and the HMBC correlations of H-1C to C-2 (*δ*_C_ 45.6), H_2_-2C to C-3 (*δ*_C_ 71.5), and C-4C (*δ*_C_ 90.5), and H-4C to C-5C (*δ*_C_ 71.9), and C-6C (*δ*_C_ 18.5)verified the presence of the sugar unit olivomycose. The NOESY spectrum further confirmed the correlations between H-1C to H-2C, H-1C to methyl proton at H-3C, H-4C to methyl proton at H-6C and established this unit with comparison of previously published literature as *β*-olivomycose.

Likewise, the unit of sugar D displayed large coupling constant (*J*_H-1D_ = 9.1 Hz) resonated at *δ*_H_ 4.62 revealed an axial orientation of H-1D and confirmed as *β*-glycosidically boundsugar. The *O*-glycodsidic connectivity of sugar C to D(C-4C-*O*-C-1D) was determined on the basis of HMBC correlations of H-4C to C-1D and H-1D to C-4C. Further, the ^1^H-^1^H COSY correlations ofH-1D/H-2D, H-2D/H-3D, H-3D/H-4D, H-4D/H-5D, and H-6D, and the HMBC cross peaks of H-1D to C-2D (*δ*_C_ 31.4) and C-3D (*δ*_C_ 28.9); H-4D to CONH_2_ (*δ*_C_ 159.6) and C-6D (*δ*_C_ 18.2)revealed the presence of sugar amicetose. The structure of **1** exhibited the substituent of a carbamoyl group at *δ*_H_ 4.24 (H-4D, dd, *J* = 10.0, 4.4 Hz) which highlighted the novelty of this compound. Therefore, sugar D was established as 4-*O*-carbamoyl-*β*-amicetose.

**Figure 4:**
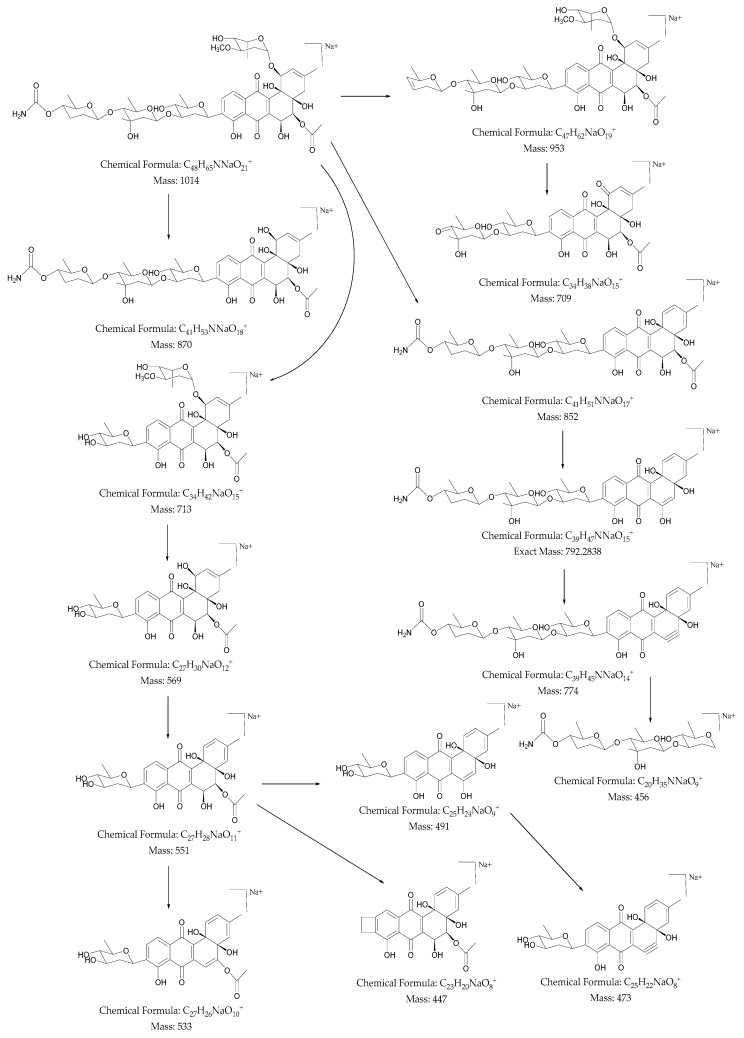
The key plausible MS*^n^* fragmentation pathway of stremycin A (**1**) was confirmed by electrospray ionization mass spectroscopy (ESI-MS/MS) analysis in positive mode.

To further confirm the new structure, ESI MS/MS fragmentation experiment of compound **1** was carried out (Figure S28). As such, the positive ion MS^n^spectrum of the structure gave the major [M + Na]^+^ ion at *m/z* 1014. As shown in Figure 4, the fragmentation of this precursor ion yielded an interesting product ion at *m/z* 953, which was attributed to the elimination of a neutral molecule CH_3_NO_2_ (61 Da) from the precursor ion at *m/z* 1014. The product ions at *m/z* 870 and 709 were generated by the loss of 144 and 161 Da, which were reasonably assigned as the elimination of C_7_H_12_O_3_ and C_7_H_15_NO_3_, respectively. Further, the fragment ion was observed at *m/z* 852, which indicated the neutral loss of 162 Da (assigned to C_7_H_14_O_4_). Similarly, the product ions at *m/z* 792, 774, and 456 were produced by the loss of 60, 18, and 318 Da, which were selected as the elimination of acetic acid (C_2_H_4_O_2_), H_2_O, and C_19_H_10_O_5_, respectively. Another major product ion peak with high intensity was observed at *m/z* 713, generated by the loss of 301 Da, specified as the elimination of C_14_H_23_NO_6_. The fragments at *m/z* 569, 551, 533, 491, and 473, which were in close agreement with the presence of *C*-glycosidic linkage at C-9 position, observed with continuous loss of 144, 18, 18, 60, and 18 Da, assigned to the removal of C_7_H_12_O_3_, H_2_O, and acetic acid (C_2_H_4_O_2_), respectively. The structure of **1**, being a new aromatic polyketide was thus termed as stremycin A.

Stremycin B (**2**) was obtained as a yellow powder. The HR-TOF-MS analysis of **2** yielded a molecular ion peak at *m/z* 1030.3860 [M + Na]^+^ (Calcd. 1030.3890) in positive mode and at *m/z* 1006.3987 [M − H]^−^ (Calcd. 1006.3925), giving the molecular formula C_48_H_65_NO_22_ (SM, Figures S29 and S30). The general feature of 1D and 2D NMR (^1^H, ^13^C, ^1^H-^1^H COSY and HMBC) spectrum (Table 2) closely resembled that of **1**, thus strongly suggesting that the structure of **2** was highly similar to the new compound **1** (Figure 1). The main difference between **1** and **2** was 16 Da, suggesting the presence of a hydroxyl group at the C-4 position in **2** instead of a methylene in **1**. According to the 1D NMR spectrum, one proton was seen at *δ*_H_ 4.17 (s, H-4), representing CH for compound **2** while two protons were seen at *δ*_H_ [1.99, 2.36 (d, *J* = 17.6 Hz, H-4)] representing CH_2_ for compound **1**. These deductions were further confirmed on the basis of the HSQC spectrum, where the cross peak notedin between the resonances of H-4 to C-4 (*δ*_C_ 70.5), and extended by HMBC correlations from H-4 to C-3, 3-Me, C-4a, and C-12b. Hence, these findings confirmed the presence of a hydroxyl group attached at C-4 to the aglycone unit of **2**. Further confirmation and analysis were carried out by ^1^H-^1^H COSY, HMBC, and NOESY experiments, where the correlations of a proton and carbon were in very close agreement to that of compound **1** and clearly confirmed the suggested structure of **2**. However, no NOESY correlation was found between H*_β_*-5 and H-4. The hydroxyl group at C-4 was determined as *β*-oriented. It was also found that the structure of **2** was highly similar to that of 4-*O*-deacetyl-warkmycin, previously reported by Helaly et al., 2015 [16], which was the synthesized version obtained under acidic conditions. Nevertheless, both structures were distinctive in sugar moieties. Finally, based on the results, the structure of **2** was elucidated as a new benz[*α*]anthracene glycoside and named as stremycin B.

Among the isolates, the known antibiotic 2-[2-(3,5-dimethyl-2-oxo-cyclohexyl)-6-oxo-tetrahydro-pyran-4yl]-acetamide (**3**) from nickel-treated extract was determined by detailed analysis of 1D NMR spectroscopy along with the comparison of data in literature [29]. Moreover, three target stress-induced compounds from the zinc treated filtrate of the same strain NA-ZhouS1 were isolated, namely, cyclo[L-(4-hydroxyprolinyl)-L-leucine] (**4**), 2-methyl-3*H*-quinazoline-4-one (**5**), and menthane derivative, 3-(hydroxymethyl)-6-isopropyl-10,12-dioxatricyclo[7.2.1.0]dodec-4-en-8-one (**6**) which were obtained and further elucidated by a detailed analysis of 1D NMR spectroscopy along with the comparison of data in literature [30,31,32].

The novel structures of **1** and **2** showed moderate antibiotic activities in comparison to the positive control tetracycline with equal MIC values of 16 µg/mL against *Pseudomonas aeruginosa*, methicillin resistant *Staphylococcus aureus* (MRSA), *Klebsiella pneumonia*, and *Escherchia coli*, while against *Bacillus subtilis*, both compounds showed the inhibition at MIC value of around 8–16 µg/mL, respectively. In earlier bioassay-guided approach it was determined that most of the angucycline related antibiotics possess moderate antibacterial activities against Gram-positive pathogens like warkmycin, chattamycin B, tetrangomycin, and vineomycin A_1_, while found mayamycin and seitomycin selective inhibit the Gram-negative pathogens [14,16,17,25,33,34]. However, all biologically active angucyclines reported previously are observed to be dependent on the length of the sugar moieties [24]. Similarly, the known antibiotic **3** exhibited antibiotic activity around 16–32 µg/mL against MRSA, *P. aeruginosa*, and *K. pneumonia*. According to previous studies, the antibiotic 2-[2-(3,5-dimethyl-2-oxo-cyclohexyl)-6-oxo-tetrahydro-pyran-4yl]-acetamide (**3**) was known to be bone resorption inhibitor and found to be an active herbicidal component [29,35,36].

## 3. Discussion

Compound **1** and **2** arestructural analogs that possessa similar polyketide aglycone, which is a tetracyclic benz[*α*]anthracene core like in angucycline-type antibioticsalong with hydrolyzable sugar units which is well known to comprise a large number of diverse representatives. As such, the structural diversity of angucyclines mainly comes from hydroxy substitution, epoxidation, and carbonyl substitutions in the region of C-4, C-5, C-6a, C-7, C-12, C-12a/C-5, C-6 or C-6a, C-12a/C-7, C12, respectively [16,28,37,38]. Moreover, other examples include the amino acid incorporations as in urdamycins and jadomycins, ring cleavages as in grincamycins and gilvocarcins, and the glycosylation at various positions, such as *O*-8 or *O*-3 and C-9 in the landomycins and saquayamycins [27,39,40,41,42]. Accordingly, the carbohydrate composition of landomycins and saquayamycins were based on the multiple trisaccharide unit’s as *β*-d-olivose-(4→1)-*β*-d-olivose-(3→1)-*α*-l-rhodinose to a said regions of the angucycline backbone. Besides, urdamycin exhibited two glycosylation positions at C-9 and 12b, while warkmycin, chattamycin B, and P371A1 displayed the *C*- and *O*-glycosylation with positions at C-9 and C-1. As such, the warkmycin antibiotic isolated from *Streptomyces* sp. Acta 2930 possessed *β*-olivose-(4→1)-*β*-olivomycose-(3→1)-*β*-amicetose at C-9 region and 4-*O*-carbamoyl-*α*-5-epi-oleandrose at the C-1 region. In the same way, the structures of **1** and **2** werealso found to possess the *C*- and *O*-glycosylated sugar units belonging to the aquayamycin-type of angucyclines with angular oxygen found in stronger relationship to warkmycinand P371A1 antibiotics. However, the compounds **1** and **2** differed from previous known compounds in the substituents of sugar moieties as *β*-olivose-(4→1)-*β*-olivomycose-(3→1)-4-*O*-carbamoyl-*β*-amicetose. Moreover, it was observed that the sites of attachment of these carbohydrates to the aglycone were the same, albeit, the structures of **1** and **2** had a carbamoyl/carbamate group at sugar D, named, 4-*O*-carbamoyl-*β*-amicetose. On the contrary, it was found that the antibiotic warkmycinhad the same group at sugar A (4-*O*-carbamoyl-*α*-*O*-5-epi-oleandrose) while antibiotic P371A1 had a ureido group at sugar C (d-*β*-amicetose). Another difference was noted in the region of aglycone at a -4 position when compared to warkmycin. The warkmycin possessed anoxygenated methine [*δ*_H_ 5.36, *δ*_C_ 68.1 (CH-4)] whereacetyl group (OAc) was present as a substituent, while **1** displayed a methylene and **2** possessed a hydroxyl group.

Many metabolites previously isolated from Streptomyces are known to be active against pathogens and display antibacterial activities which are desperately needed on the front line in combating microbial infections. Due to the increasing threat of antibiotic resistance pathogens, scientists are urged to focus on the isolation of more antimicrobial compounds along with the investigation of their mechanisms of action and biosynthetic pathways. As illustrated in this study, heavy metals being applied as elicitors, here referring to the heavy metal nickel ion revealed a distinct HPLC guided profile when compared to the normal one, showing an influence on the secondary metabolome of the *Streptomyces pratensis* strain NA-ZhouS1. We hypothesize that these results are indicative of cryptic gene cluster activation consequential to the metal stress imposed on the strain under study, ultimately resulting in the production of two novel compounds with activity against pathogenic bacterial isolates. It was also observed that the normal products displayed in the untreated culture were considerably lowered when stressed by metals (Figure 2), showing that nickel ion not only stimulates a nonactivated biosynthetic pathway but also impacts the normal biosynthetic capabilities of the strain. We observed that normal growth of *S. pratensis* was repressed when the nickel ion concentration reached around 800 µM, likely having a global effect on processes which occur under normal condition. Moreover, research is required to scrutinize more effective elicitors or ways/techniques for elicitation of cryptic genes clusters of marine microbes may lead to the production of unexpected, albeit potentially potent natural products.

## 4. Materials and Methods

### 4.1. General Experimental Procedures

Electrospray ionization mass spectrometry (ESIMS) were recorded on an Agilent 6460 Triple Quade Liquid Chromatography with Mass Spectrometry (Agilent, Beijing, China). HPLC analysis used was composed of a Waters 717 plus Autosampler, a Waters 600 Controller, a Waters 996 Photodiode Array Detector and a Waters Millog workstation (Waters, Shingawa, Tokyo, Japan), while preparative HPLC was performed on an Agilent-1100 system (ChuangXintongheng, Beijing, China) equipped with a Venusil MP-C18 column (10 mm × 250 mm, Agila Technologies, Tianjin, China). Reverse phase column chromatography was performed. ^1^H NMR (recorded on 500 MHz), ^13^C NMR (recorded on 125 MHz), DEPT-135, ^1^H-^1^H COSY, HMQC, HMBC, and NOESY spectra were measured at 25 °C on a Bruker ADVANCE DMX 500 NMR spectrometer with TMS as internal standard (Bruker, Fällanden, Switzerland). Methanol was used as solvent for NMR experiments. The organic solvents used in chromatographic separation were of analytical grade purchased from Sayfo Technology (Tianjin, China) and chromatographic grade for HPLC analysis purchased from Tedia, Fairfield, OH, USA. Deionized water was prepared by Reverse osmosis Milli-Q water (18 MW) (Millipore, Bedford, MA, USA) and used for all solutions and dilutions. Agar powder for plate culture and other heavy metals including nickel (NiCl_2_·6H_2_O) were purchased from Sinopharm Chemical Reagent Co., Ltd. (Shanghai, China).

### 4.2. Isolation and Identification of Streptomyces sp. NA-ZhouS1

The strain NA-ZhouS1 was isolated from a marine sediment sample, collected from the East China Sea, Zhoushan. The plate dilution method was used to isolate actinomycetes from the sample suspension. Approximately 0.5–1 g of each fresh sediment sample was directly inoculated into the presterilized glass tubes and diluted with artificial sea water. Serially diluted samples were plated in the gauze’s (GS), starch casein nutrient (SCN) and Aspergillus minimal (AMM) agar medium in triplicate. All the plates were supplemented with nystatin (0.05 g/L) to prevent fungal contamination. The plates were incubated at 28 °C and actinomycete colonies counted from the 7th day onwards up to the 25th days. Single colony of actinomycete was picked up and grown separately for inoculation an agar slant containing the same isolation medium. 16S ribosomal DNA gene was used to identify the strain. The strain NA-ZhouS1 showed 99.93% resemblance to *Streptomyces pratensis*. Sequences were then searched by online database listed in (SM, Table S29). This species was found with off-white to grey aerial spores on gauze’s medium and carried smooth-surfaced spores in straight or flexuous spore chains. This actinomycete sp. was previously known as *Streptomyces flavogriseus*, but then was reclassified as *Streptomyces pratensis* [43,44]. A neighbor-joining tree was constructed using software package of Molecular Evolutionary Genetics Analysis (MEGA), version 7.0, Pennsylvania State University, United States for further phylogenetic analysis (SM, Figure S32).

### 4.3. Analysis of Normal Culture and Metal Stress Cultivation

For screening and initial analysis of normal culture, the spores of NA-ZhouS1 strain were inoculated in 500-mL Erlenmeyer flasks containing 200-mL liquid Gauze’s medium (20 g soluble starch, 1 g KNO_3_, 0.5 g K_2_HPO_4_, 0.5 g MgSO_4_·7H_2_O, 0.01 g FeSO_4_·7H_2_O, 35 g sea salt per liter at pH 7.4) and was grown on a rotatory shaker at 180 rpm for 7 days at 28 °C. Afterwards, the same actinomycete strain NA-ZhouS1 was stressed under different metal conditions like cobalt (CoCl_2_·6H_2_O), nickel (NiCl_2_·6H_2_O), zinc (ZnSO_4_·7H_2_O), chromium (CrCl_3_·6H_2_O), and manganese (MnCl_2_·6H_2_O), while each metal was applied with four different concentrations of 100, 200, 400, and 800 µM, respectively. The mycelium was removed and the filtrate was extracted twice with an equal volume of ethyl acetate (EtOAc). Finally, extracts were subjected to analytical reversed phase HPLC-UV for further screening by comparing treated and untreated extracts. Consequently, the comparison of the RP-HPLC profiles of the extracts from the strain NA-ZhouS1 revealed the formation of new metabolites following use of 100 µM nickel ion (NiCl_2_·6H_2_O), and thus grabbed our attention as a strong contributing factor toward activation of cryptic gene clusters. Additionally, the extract of both normal and stressed cultures, were assayed after overnight incubation at 37 °C for their antibacterial capacities which boost up our judgment to enlarge 100 µM nickel ion (NiCl_2_·6H_2_O) culture due to its effective inhibitory abilities.

### 4.4. Large Scale Fermentation, Extraction and Isolation

The strain NA-ZhouS1 was cultured in the presence of 100 µM nickel treated agent for extraction into 500-mL Erlenmeyer flasks in 200 mL liquid gauze’s medium. A total of 25 L fermentation containing 100 µM nickel ions was carried out at 28 °C on a rotary shaker at 180 rpm for 10 days. Thereafter, the fermentation broth was combined and filtered. Subsequently, the filtrate was extracted with (EtOAc) ethyl acetate (2 × 200 mL) twice and dried in vacuo, to provide an organic extract of (3 g).

The crude extract (3 g) was filtered and dissolved in methanol. The extract was then subjected to silica gel column (reverse phase column), using MeOH-H_2_O as an eluent at the ratio of (20:80 → 100:00) to yield 8 fractions. As such, the main fractions obtained were dissolved in methanol and centrifuged at 12,000 rpm for 10 min. The first major fraction was further subjected to preparative HPLC (flow rate 10 mL/min, UV detector 210 nm Ruijia company, Hangzhou, China), using MeOH-H_2_O as an eluent, to yielded compound **1** (6.1 mg, 60:40, *t*_R_ 22 min) and compound **2** (7.3 mg, 60:40, *t*_R_ 21 min). The second yielded fraction was further purified by preparative HPLC (flow rate 10 mL/min, UV detector 210 nm), using MeOH-H_2_O as an eluent, to give a known antibiotic **3** (4.5 mg), previously isolated from a soil *Streptomyces* sp. SPRI-70014 and SANK 61296. Similarly, three more known compounds **4**, **5**, and **6** were isolated from the zinc treated (100 µM) crude extract of the same strain using analytical HPLC [flow rate 0.8 mL/min, eluted mode 0~30 min 20%~100% (H_2_O/MeOH), 30–50 min 100% MeOH, UV detector 210 nm].

Stremycin A (**1**): Yellow powder; ^1^H NMR and ^13^C NMR, see Table 1; HR-TOF-MS *m/z* 1014.3937 [M + Na]^+^ (Calcd. for C_48_H_65_NNaO_21_, 1014.3941).

Stremycin B (**2**): Yellow powder; ^1^H NMR and ^13^C NMR, see Table 2; HR-TOF-MS *m/z* 1030.3860 [M + Na]^+^ (Calcd. for C_48_H_65_NNaO_22_, 1030.3890).

### 4.5. Antimicrobial Activity of Stressed Metabolites

Microbial activity was assessed using the conventional broth dilution assay with Gram-positive and Gram-negative clinical pathogens, namely, *K. pneumoniae* [CMCC (B) 46117], methicillin resistant *S. aureus* (MRSA), *B. subtilis* [CMCC(B) 63501], *E. coli* [CMCC(B) 44102], and *P. aeruginosa* [CMCC(B) 10104]. These pathogens were cultured in nutrient agar medium and left overnight incubation at 37 °C for 12–18 h. Each pathogenic culture was then diluted in 0.9% saline to an inoculum density of 5 × 10^5^ cfu by comparison with a McFarland standard. Tetracycline was used as positive control while the solvent methanol was used as negative control. Methanolic solution of 3-[4,5-dimethylthiazol-2-yl]-2,5-diphenyltetrazolium bromide (MTT; Lancaster, PA, USA) was used to observe pathogenic growth by a change in color. 125 µL Muller Hinton broth was distributed into the 96-well plates. Similarly, samples were dispensed into well 1 and serially diluted across the well followed bacterial inoculation. Finally, the plates were incubated at 37 °C for 18 h and the results for bacteriostatic abilities of the compounds were noted in triplicate as MICs.

## Figures and Tables

**Table 1:** NMR spectrum data for stremycin A (**1**), ^1^H NMR (500 MHz, *δ* in ppm), ^13^C NMR (125 MHz, *δ* in ppm) in MeOD.

Position	*δ*_C_, Type	*δ*_H_ (Mult., *J* in Hz)	HMBC	COSY
1	82.1, CH	4.34, d (4.2)	C-1A, C-2, C-12b, C-4a	H-2
2	120.8, CH	5.63, s	C-3	H-1
3	136.6, C	-	-	-
4	36.3, CH_2_	1.99, d (17.6); 2.36, d (17.6)	Me-3, C-4a	-
4a	75.6, C	-	---	-
5	76.2, CH	5.84, d, (6.8)	C-6, COMe	H-6
6	69.9, CH	4.92, d, (6.8)	C-5, C-6a, C-12a	H-5
6a	145.2, C	-	-	-
7	190.5, C	-	-	-
7a	115.6, C	-	-	-
8	158.6, C	-	-	-
9	139.3, C	-	-	-
10	134.2, CH	7.87, d (7.9)	C-7a, C-8, C-1B, C11a	H-11
11	120.1, CH	7.64, d (7.9)	C-7a, C-9, C-12	H-10
11a	132.6, C	-	-	-
12	187.2, C	-	-	-
12a	146.0, C	-	-	-
12b	78.5, C	-	-	-
3-Me	23.5, CH_3_	1.68, s	C-3, C-4	-
5-COMe	172.3, C	-	-	-
	20.9, CH_3_	2.20, s	-	-
**Sugar A**				
1A	100.6, CH	4.60, d (4.0)	C-1, C-3A, C-5A	H-2A
2A	30.6, CH_2_	1.31, overlapped; 1.88, m	-	H-1A, H-3A
3A	78.4, CH	3.29, m	OMe-3A	H-4A
4A	66.9, CH	3.49, m	-	H-5A
5A	63.4, CH	4.29, m	-	H-6A
6A	16.8, CH_3_	1.18, d (6.6)	C-5A	H-5A
OMe-3A	57.5,C	3.24, s	-	-
**Sugar B**				
1B	72.4, CH	4.89, s	C-10, C-9, C-8, C7a, C-2B, C-3B, C-4B, C-5B	H-2B
2B	38.6, CH_2_	1.42, d (12.6);2.50, dd (12.1, 4.2)	-	H-1B, H-3B
3B	82.5, CH	3.84, m	C-1C	H-4B
4B	76.8, CH	3.11, t (8.9)	-	H-5B
5B	77.7, CH	3.46, m	C-6B	H-6B
6B	18.8, CH_3_	1.38, d (6.1)	-	-
**Sugar C**				
1C	99.3, CH	4.78, dd (9.9, 1.8)	C-3B, C-2C	H-2C
2C	45.6, CH_2_	1.68, overlapped; 1.95, m	C-3C, C-4C	H-1C
3C	71.5, C	-	-	-
4C	90.5, CH	3.19, d (9.6)	C-5C, C-6C, C-1D	H-5C
5C	71.9, CH	3.55, m	-	H-6C
6C	18.5, CH_3_	1.31, d (6.1)	C-5C	H-5C
Me-3C	22.6, CH_3_	1.25, s	C-3C, C-4C	-
**Sugar D**				
1D	104.5, CH	4.62, s	C-4C, C-2D, C-3D	H-2D
2D	31.4, CH_2_	1.64, m; 1.99, overlapped	-	H-3D
3D	28.9, CH_2_	1.57, m; 2.14, m	-	H-4D
4D	73.9, CH	4.24, dd (10.0, 4.4)	C-6D, CONH_2_	H-5D
5D	75.3, CH	3.65, m	-	H-6D
6D	18.2, CH_3_	1.22, d (6.2)	C-5D	-
4D-CONH_2_	159.6, C	-	C-4D	-

**Table 2:** NMR spectrum data for stremycin B (**2**), ^1^H NMR (500 MHz, *δ* in ppm), ^13^C NMR (125 MHz, *δ* in ppm) in MeOD.

Position	*δ*_C_, Type	*δ*_H_ (Mult., *J* in Hz)	HMBC	COSY
1	81.8, CH	4.43, d (3.8)	C-1A, C-2, C-3, C-12b, C-4a	H-2
2	123.1, CH	5.72, d (3.2)	3-Me, C-4	H-1
3	138.7, C	-	-	-
4	70.5, CH	4.17, s	3-Me, C-3, C-4a, C-12b	-
4a	76.2, C	-	-	-
5	75.9, CH	5.76, d (6.8)	C-6, COMe	H-6
6	69.8, CH	4.91, m (6.8)	C-5, C-6a, C-12a	H-5
6a	144.5, C	-	-	-
7	190.8, C	-	-	-
7a	115.6, C	-	-	-
8	158.7, C	-	-	-
9	139.4, C	-	-	-
10	134.2, CH	7.85 d (7.7)	C-8, C-1B, C11a	H-11
11	120.0, CH	7.61, d (7.7)	C-7a, C-9, C-12	H-10
11a	132.5, C	-	-	-
12	187.3, C	-	-	-
12a	146.7, C	-	-	-
12b	79.7, C	-	-	-
3-Me	21.9, CH_3_	1.95, s	C-3, C-4	-
5-COMe	173.0, C	-	-	-
	20.6, CH_3_	2.02, s	-	-
**Sugar A**				
1A	100.9, CH	4.57, d (4.4)	C-1, C-3A, C-5A	H-2A
2A	30.3, CH_2_	1.32, overlapped; 1.85, m	C-1A	H-1A, H-3A
3A	78.4, CH	3.27, m	C-4A	H-4A
4A	67.5, CH	3.47, m	-	H-5A
5A	63.7, CH	4.10, d (6.5)	-	H-6A
6A	16.8, CH_3_	1.17, d (6.6)	C-5A	H-5A
OMe-3A	57.7, C	3.30, s	-	-
**Sugar B**				
1B	72.4, CH	4.87, m (overlapped)	C-9, C-10	H-2B
2B	38.6, CH_2_	1.42, m; 2.49, dd (12.3, 4.6)	C-3B, C-4B	H-1B, H-3B
3B	82.3, CH	3.84, m	C-4B, C-1C	H-4B
4B	76.9, CH	3.11, dd (11.1, 6.7)	C-3B, C-5B, C-6B	H-5B
5B	77.7, CH	3.44, dd (6.9, 3.5)	C-6B	H-6B
6B	18.8, CH_3_	1.37, d (6.1)	C-5B	-
**Sugar C**				
1C	99.3, CH	4.77, d (9.9)	C-3B, C-2C	H-2C
2C	45.6, CH_2_	1.95, overlapped; 1.67, m	C-3C, C-4C	H-1C
3C	71.5, C	-	-	-
4C	90.5, CH	3.18, d (9.6)	Me-3C, C-5C, C-6C, C-1D	H-5C
5C	72.0, CH	4.81, d (6.5)	---	H-6C
6C	18.5, CH_3_	1.30, d (6.1)	C-5C	H-5C
Me-3C	22.6, CH_3_	1.25, s	C-3C, C-4C	-
**Sugar D**				
1D	104.5, CH	4.61, d (9.2)	C-4C, C-2D, C-3D	H-2D
2D	31.4, CH_2_	1.99, m; 1.63, m	-	H-3D
3D	28.9, CH_2_	2.14, m; 1.59, m	-	H-4D
4D	73.9, CH	4.24, dd (9.7, 5.5)	C-6D, CONH_2_	H-5D
5D	75.3, CH	3.62, m	-	H-6D
6D	18.2, CH_3_	1.22, d (6.1)	C-5D	-
4D-CONH_2_	159.6, C	-	C-4D	-

## Supplementary Materials

The following are available online at http://www.mdpi.com/1660-3397/16/9/331/s1, Figures S1, S9, S16, S19, S21 and S24: ^1^H NMR for compounds **1**, **2**, **3**, **4**, **5**, and **6**. Figures S2, S10, S17, S20, S22 and S25: ^13^C NMR for compounds **1**, **2**, **3**, **4**, **5**, and **6**. Figures S3, S11, S18 and S23: DEPT135 for compounds **1**, **2**, **3** and **5**. Figures S4 and S12: COSY for compounds **1** and **2**. Figures S5 and S13: HMQC for compounds **1** and **2**. Figures S6, S7 and S15: HMBC for compounds **1** and **2**. Figures S8 and S15: NOESY for compounds **1** and **2**. Figures S26, S27, S29 and S30: HR-TOF-MS in positive and negative mode for compounds **1** and **2**. Figure S28: MS*^n^* spectrum of compound **1**. Figure S31: 16S ribosomal DNA gene, full sequence of *Streptomyces* sp. (strain NA-ZhouS1). Figure S32: Neighbor-joining phylogenetic tree based on 16S rDNA sequence for strain NA-ZhouS1. Table S1: Biological activities of compounds **1**–**5** (MIC values are given in µg/mL).
