# Supplementary material for: Stress-Driven Discovery of New Angucycline-Type Antibiotics from a Marine Streptomyces pratensis NA-ZhouS1
Source: Mar Drugs. 2018 Sep 12;16(9):331. doi: 10.3390/md16090331 (PMC6163593; doi:10.3390/md16090331)
Supplement: Supplementary file 1 [file marinedrugs-16-00331-s001.pdf]

Supplementary Material

Stress-Driven Discovery of New Angucycline-Type Antibiotics from a Marine *Streptomyces pratensis* NA-ZhouS1

Najeeb Akhter [1], Yaqin Liu [2], Bibi Nazia Auckloo [1], Yutong Shi [1], Kuiwu Wang [3], Juanjuan Chen [4], Xiaodan Wu [5], and Bin Wu [1,*]

[1] Ocean College, Zhejiang University, Hangzhou 310058, China. na_memon@yahoo.com; naz22ia@hotmail.com; 11434028@zju.edu.cn; wubin@zju.edu.cn
[2] Department of Chemistry, Zhejiang University, Hangzhou 301000, China. yaqin86@zju.edu.cn
[3] Department of Chemistry, Zhejiang Gongshang University, Hangzhou 310012, China. wkwnpc@zjgsu.edu.cn
[4] Key Laboratory of Applied Marine Biotechnology, Ningbo University, Chinese Ministry of Education, Ningbo 315211, China. chenjuanjuan@nbu.edu.cn.
[5] Centre of Analysis and Measurement, Zhejiang University, Hangzhou 310058, China. wxd_zju@163.com
* Correspondence: wubin@zju.edu.cn; Tel. /Fax: +86-05802092258

List of supporting information

**Figure S1**	^{1}H NMR spectrum in MeOD for compound **1**	S3
**Figure S2**	^{13}C NMR spectrum in MeOD for compound **1**	S3
**Figure S3**	DEPT-135 spectrum in MeOD for compound **1**	S4
**Figure S4**	^{1}H-^{1}H COSY spectrum in MeOD for compound **1**	S4
**Figure S5**	HSQC spectrum in MeOD for compound **1**	S5
**Figure S6**	HMBC spectrum in MeOD for compound **1**	S5
**Figure S7**	HMBC-2 spectrum in MeOD for compound **1**	S6
**Figure S8**	NOESY spectrum in MeOD for compound **1**	S6
**Figure S9**	^{1}H NMR spectrum in MeOD for compound **2**	S7
**Figure S10**	^{13}C NMR spectrum in MeOD for compound **2**	S7
**Figure S11**	DEPT-135 spectrum in MeOD for compound **2**	S8
**Figure S12**	^{1}H-^{1}H COSY spectrum in MeOD for compound **2**	S8
**Figure S13**	HSQC spectrum in MeOD for compound **2**	S9
**Figure S14**	HMBC spectrum in MeOD for compound **2**	S9
**Figure S15**	NOESY spectrum in MeOD for compound **2**	S10
**Figure S16**	^{1}H NMR spectrum in MeOD for compound **3**	S10
**Figure S17**	^{13}C NMR spectrum in MeOD for compound **3**	S11
**Figure S18**	DEPT-135 spectrum in MeOD for compound **3**	S11
**Figure S19**	^{1}H NMR spectrum in $CDCl_3$ for compound **4**	S12
**Figure S20**	^{13}C NMR spectrum in $CDCl_3$ for compound **4**	S12
**Figure S21**	^{1}H NMR spectrum in $CDCl_3$ for compound **5**	S13
**Figure S22**	^{13}C NMR spectrum in $CDCl_3$ for compound **5**	S13
**Figure S23**	DEPT-135 spectrum in $CDCl_3$ for compound **5**	S14
**Figure S24**	^{1}H NMR spectrum in $CDCl_3$ for compound **6**	S14
**Figure S25**	^{13}C NMR spectrum in $CDCl_3$ for compound **6**	S15
**Figure S26**	HR-TOF-MS spectrum in negative mode for compound **1**	S15
**Figure S27**	HR-TOF-MS spectrum in positive mode for compound **1**	S15
**Figure S28**	MS^n spectrum of compound **1**	S16
**Figure S29**	HR-TOF-MS spectrum in negative mode for compound **2**	S16
**Figure S30**	HR-TOF-MS spectrum in positive mode for compound **2**	S17
**Figure S31**	16S ribosomal DNA gene, full sequence of *Streptomyces* sp. NA-ZhouS1	S17
**Figure S32**	Neighbor-joining phylogenetic tree based on 16S rDNA sequence for strain NA-ZhouS1	S18
**Table S1**	Biological activities of compounds (**1**-**5**) (MIC values are given in μg/mL)	S17

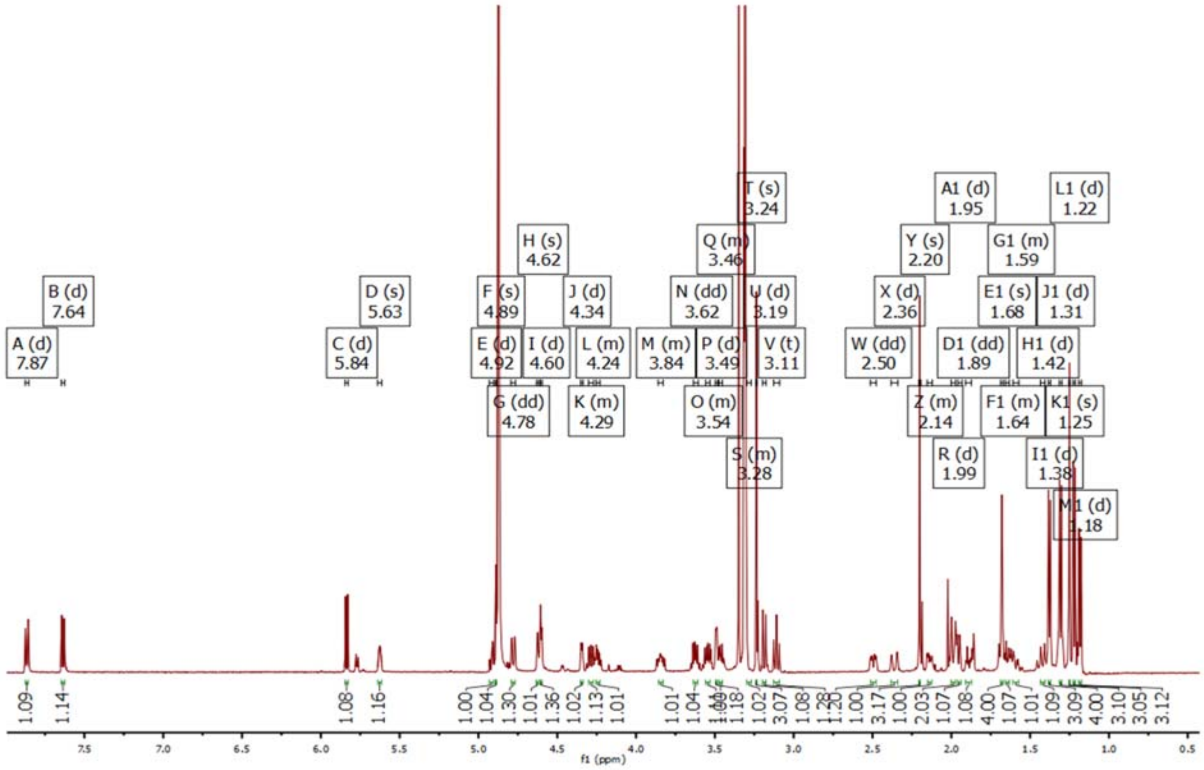

Figure S 1. ^{1}H NMR spectrum in MeOD for compound **1**.

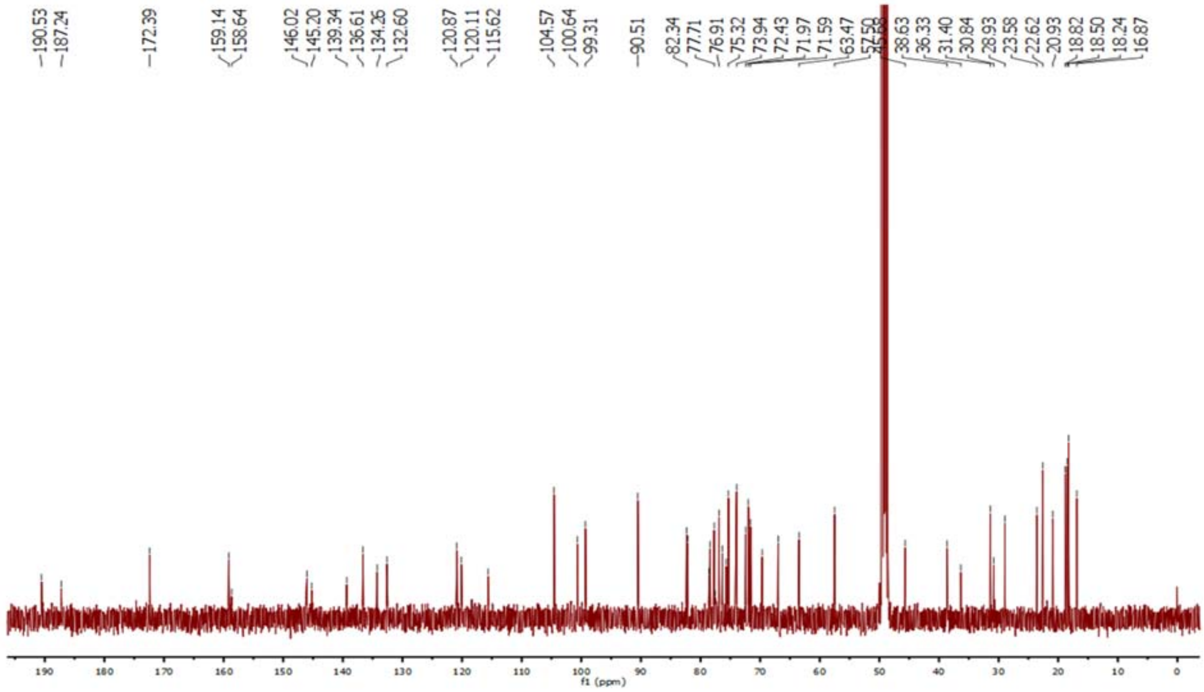

Figure S 2. ^{13}C NMR spectrum in MeOD for compound **1**.

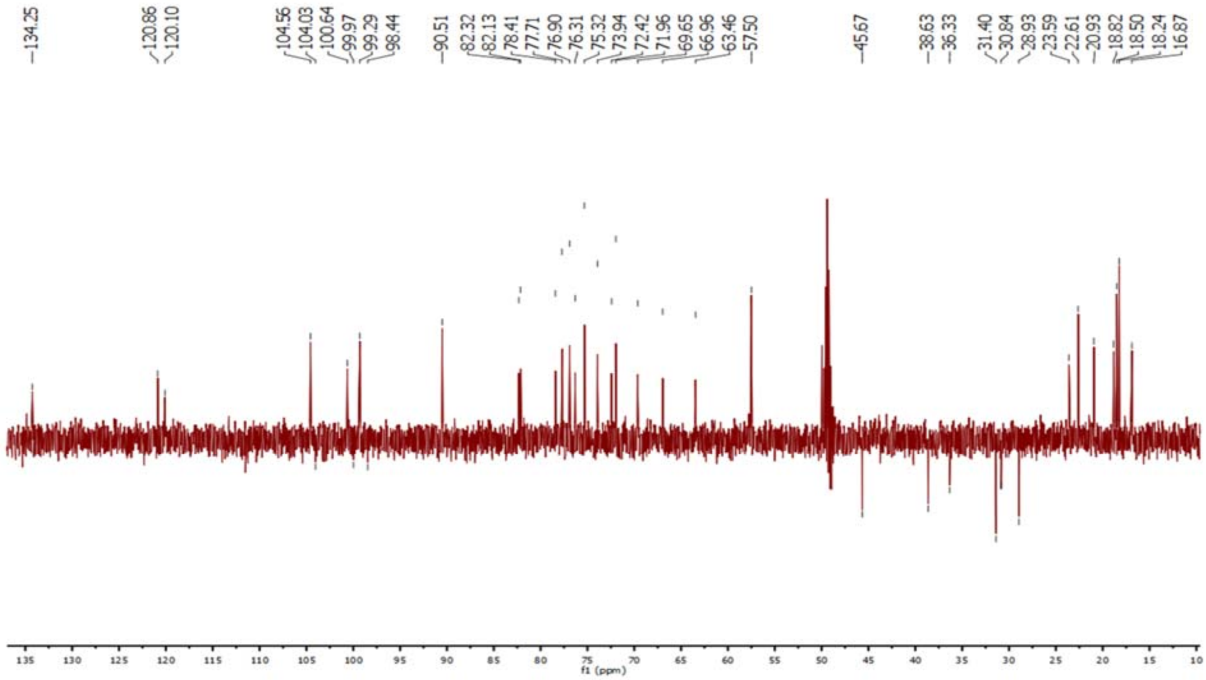

Figure S 3. DEPT-135 spectrum in MeOD for compound **1**.

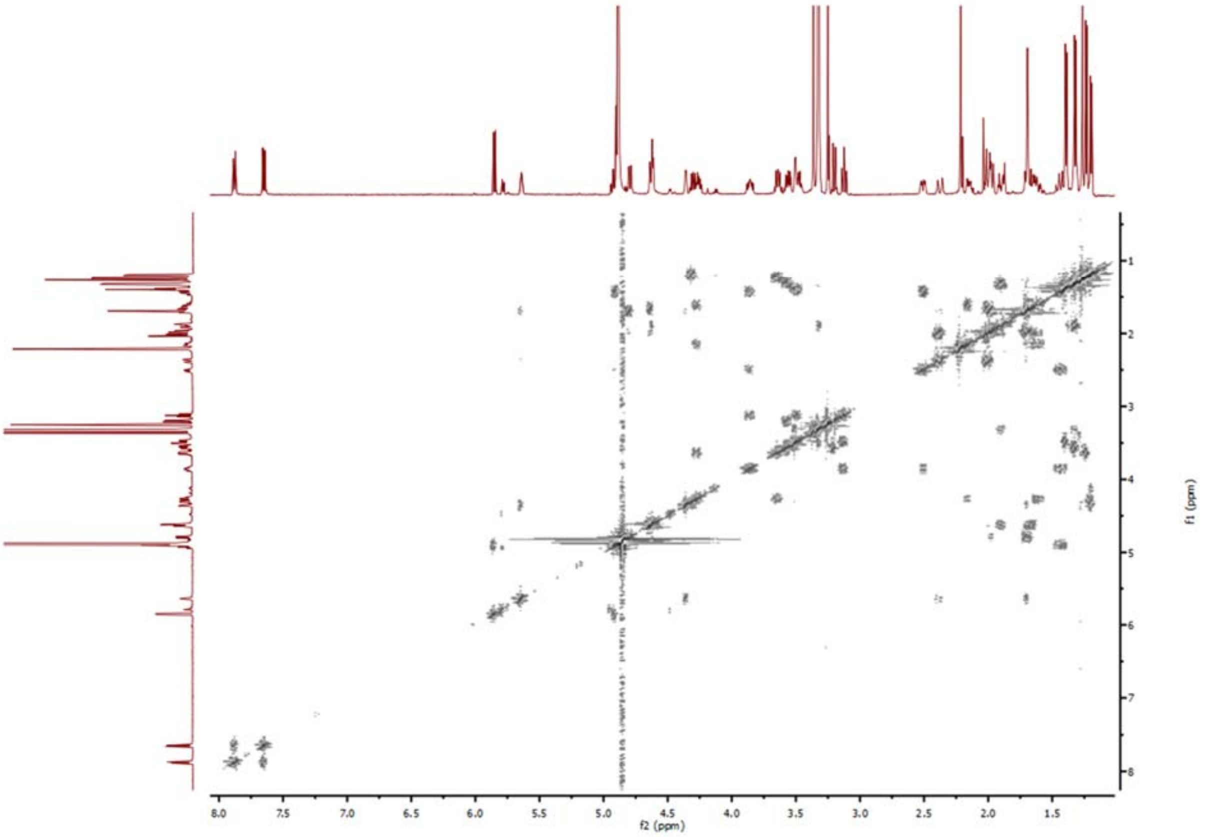

Figure S 4. COSY spectrum in MeOD for compound **1.**

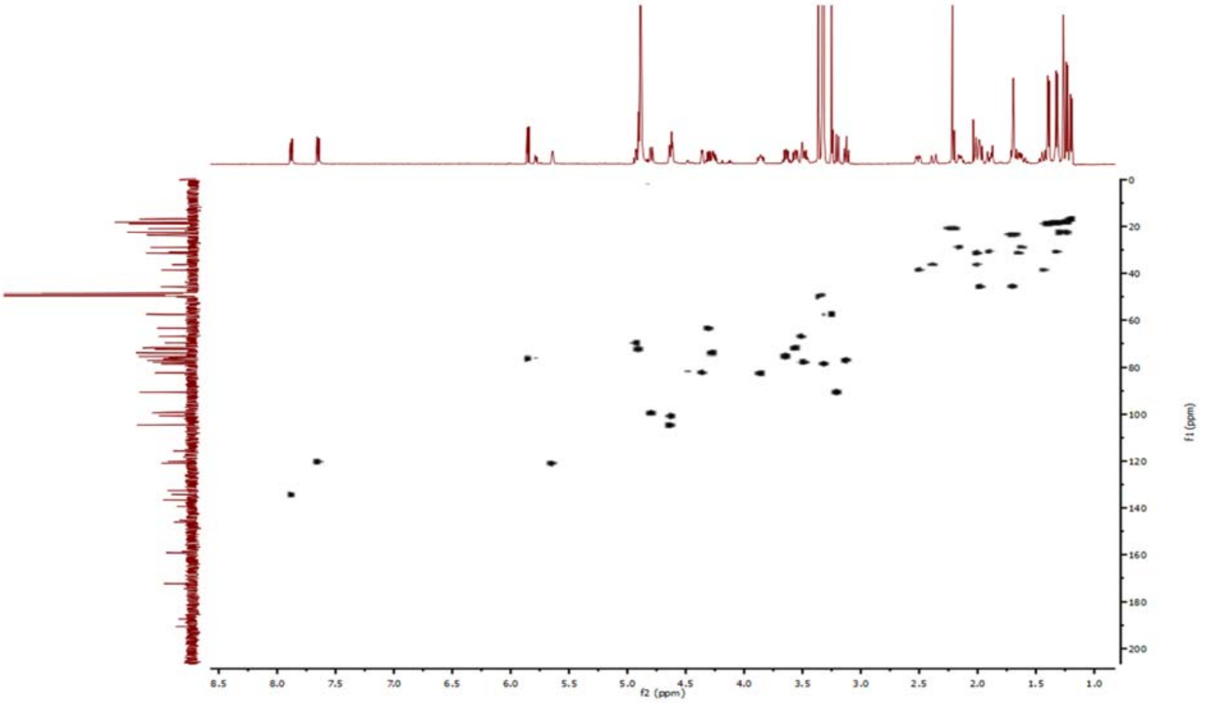

Figure S 5. HSQC spectrum in MeOD for compound **1**.

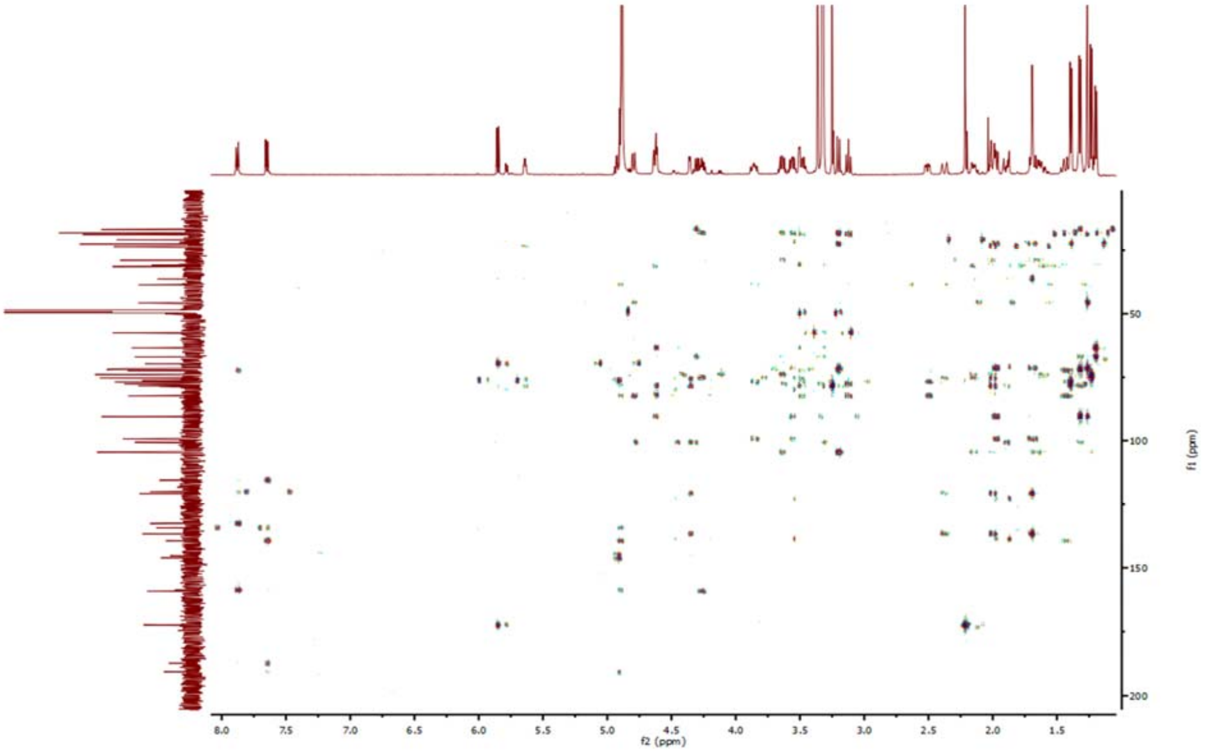

Figure S 6. HMBC spectrum in MeOD for compound **1**.

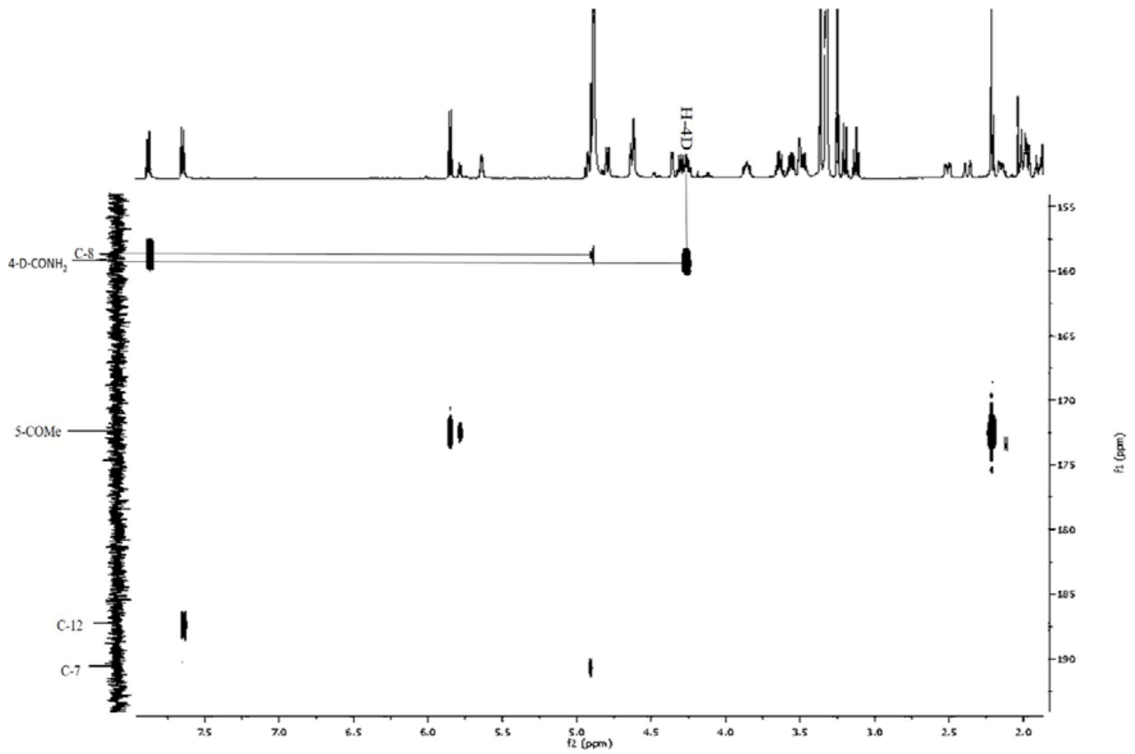

Figure S 7. HMBC-2 spectrum in MeOD for compound **1**. Enlarged view of the spectrum to clarify the cross peaks of H-4D to carbamoyl carbon resonated at δc 159.6.

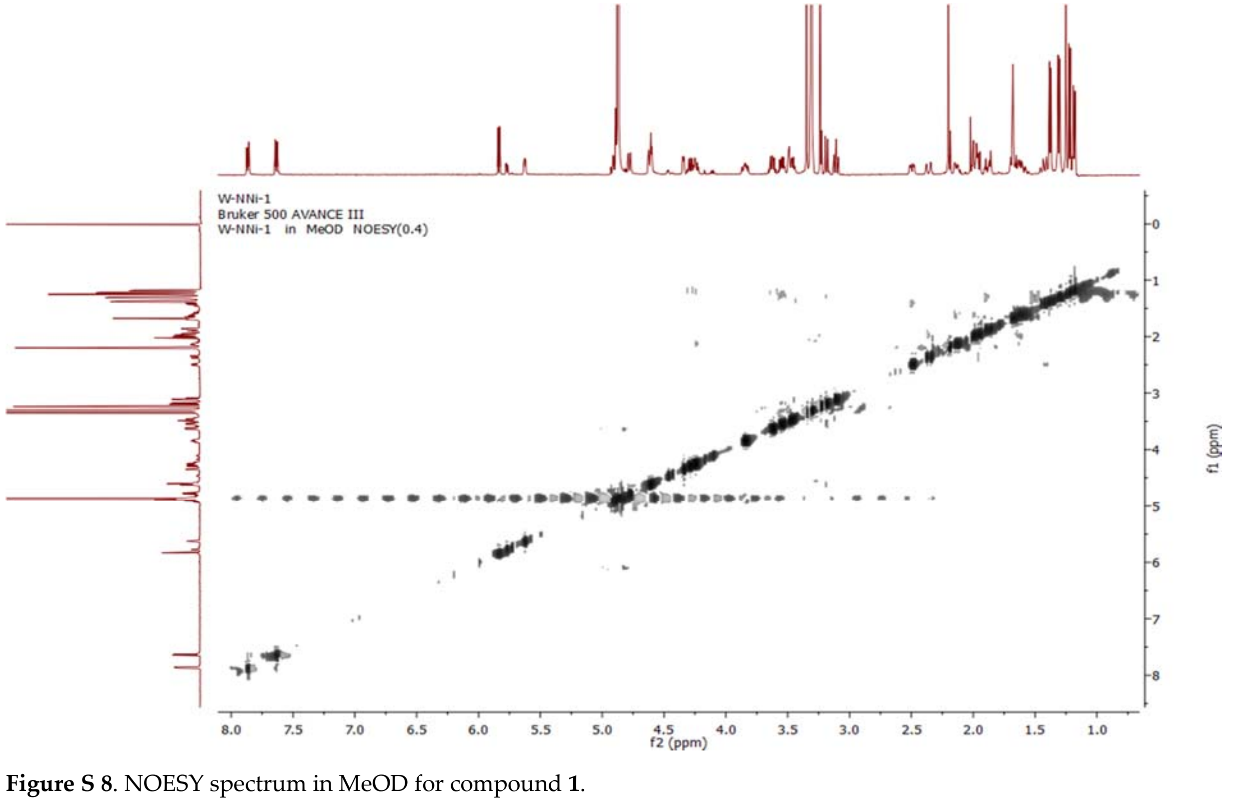

Figure S 8. NOESY spectrum in MeOD for compound **1**.

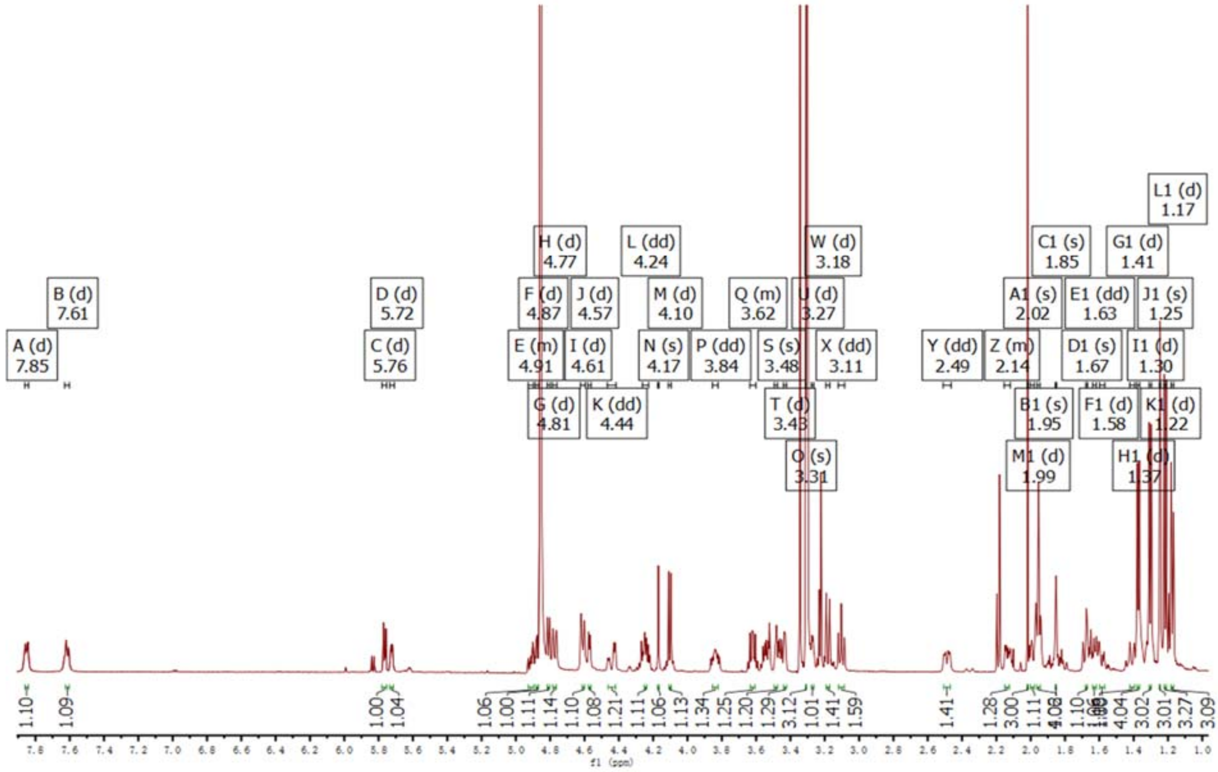

Figure S 9. ^{1}H NMR spectrum in MeOD for compound **2**.

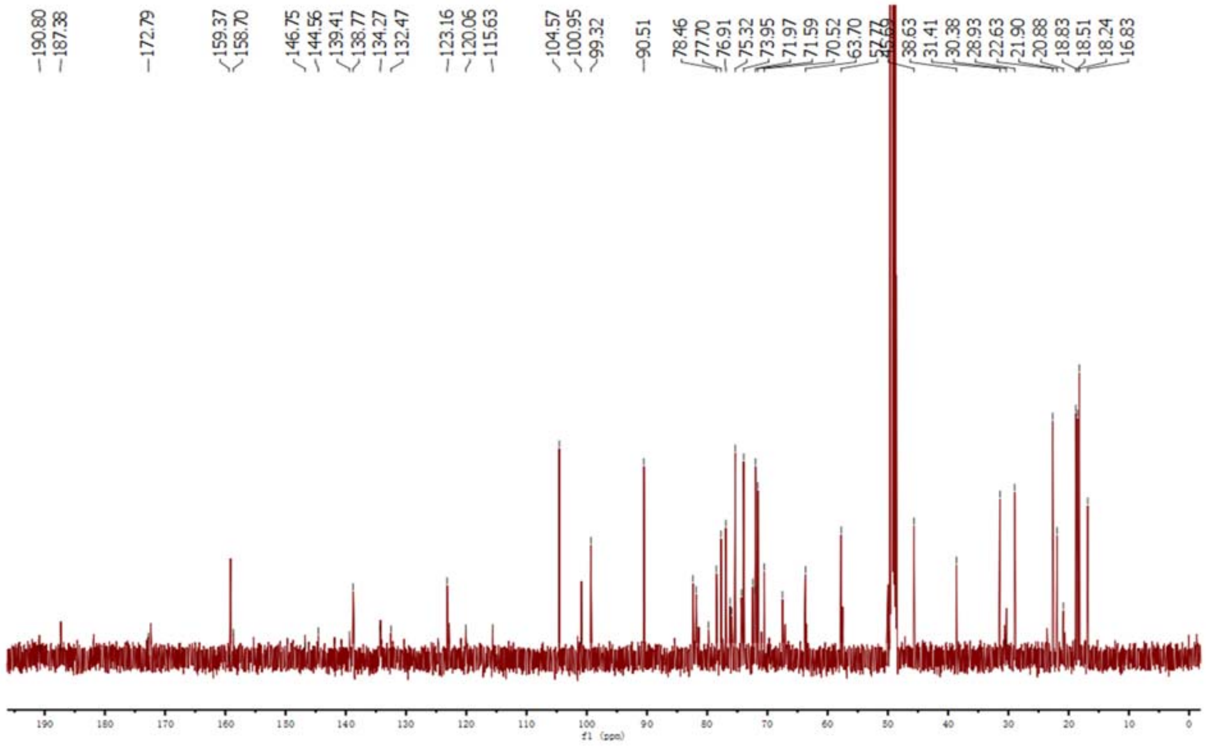

Figure S 10. ^{13}C NMR spectrum in MeOD for compound **2**.

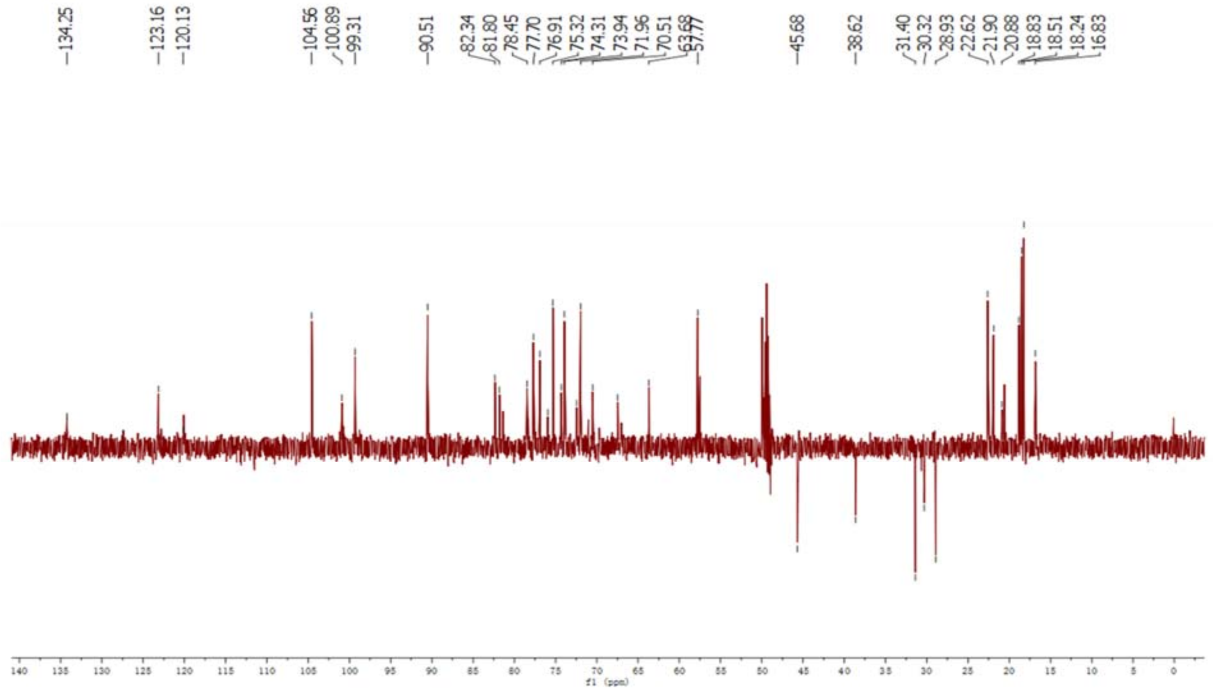

Figure S 11. DEPT-135 spectrum in MeOD for compound **2.**

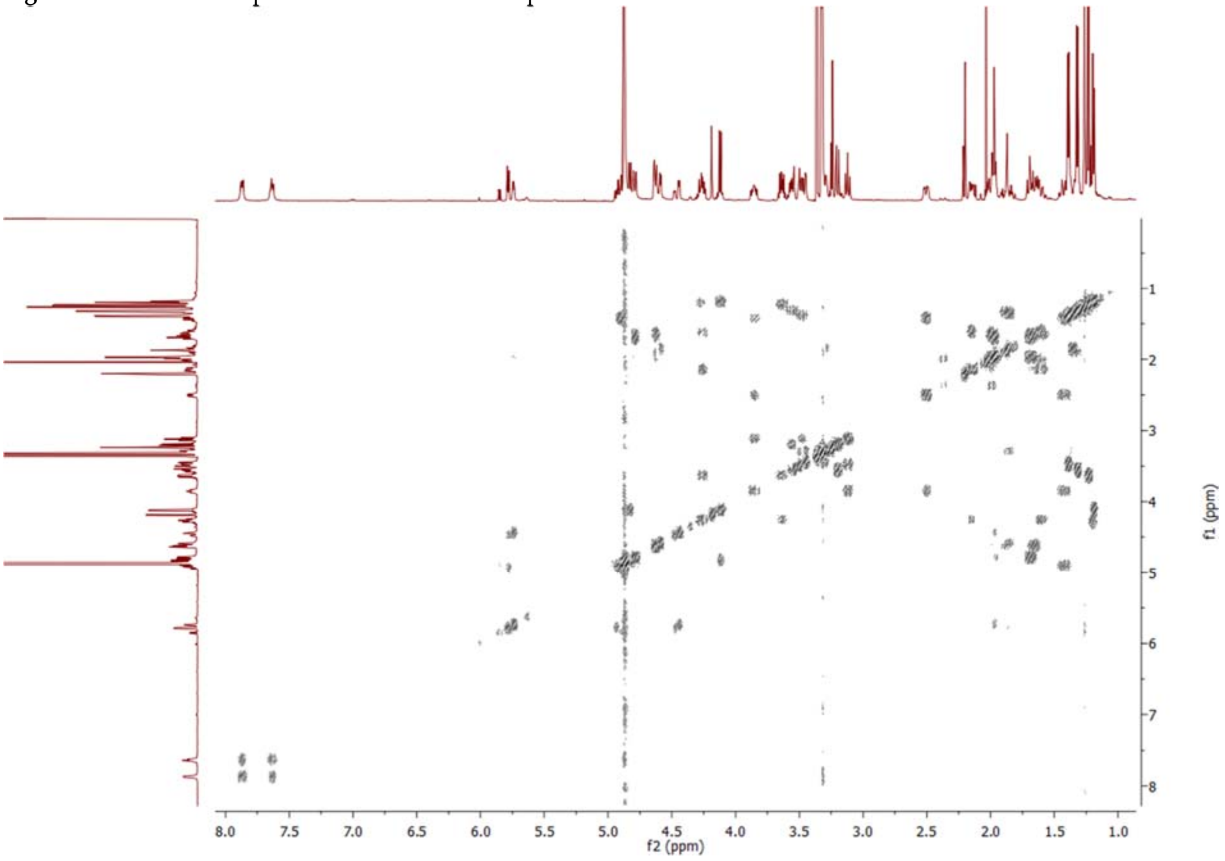

Figure S 12. COSY spectrum in MeOD for compound **2**.

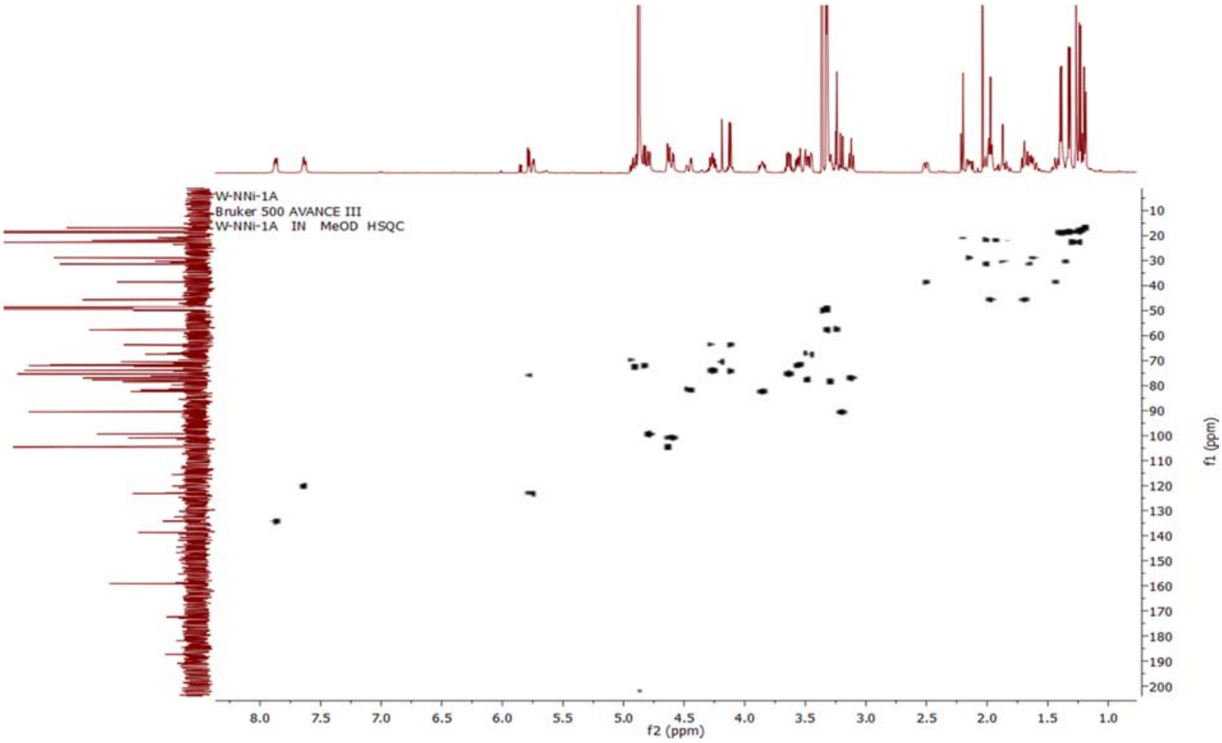

Figure S 13. HSQC spectrum in MeOD for compound **2**.

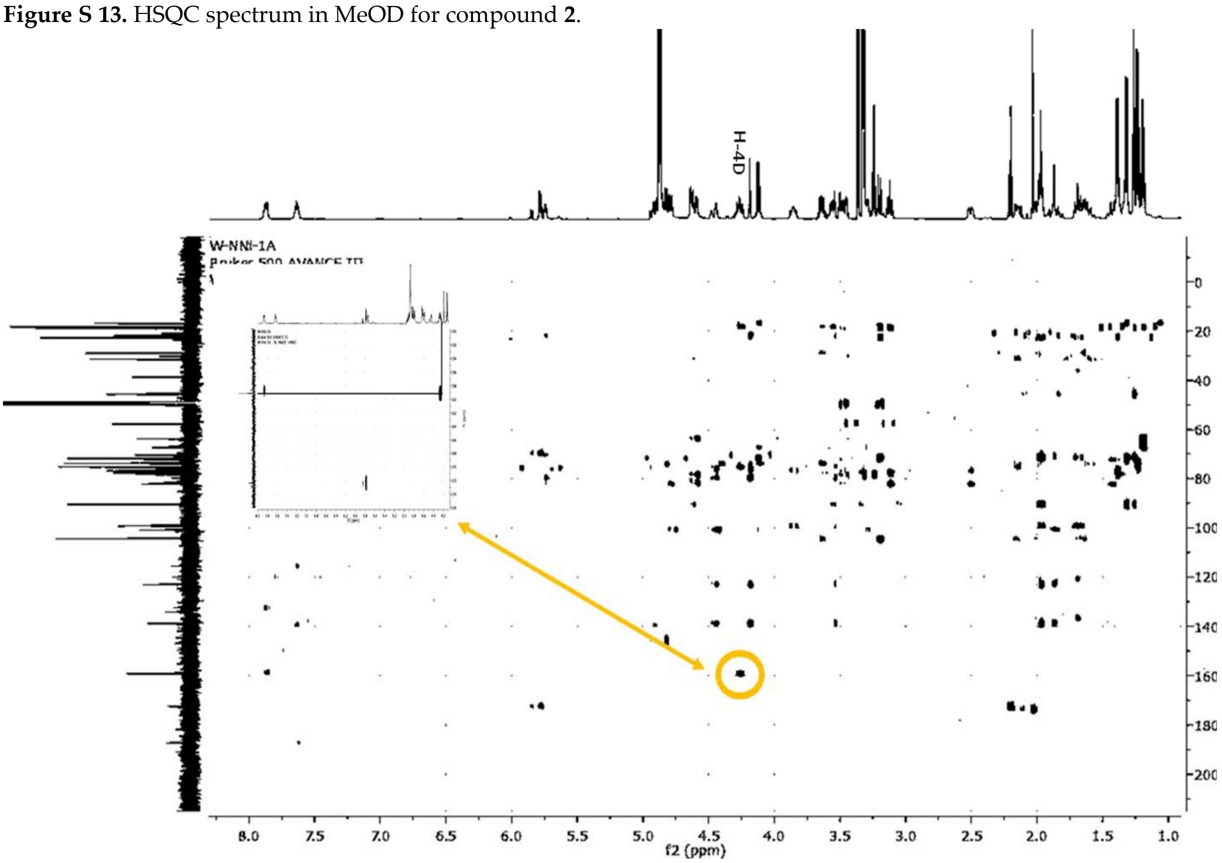

Figure S 14. HMBC spectrum in MeOD for compound **2**.

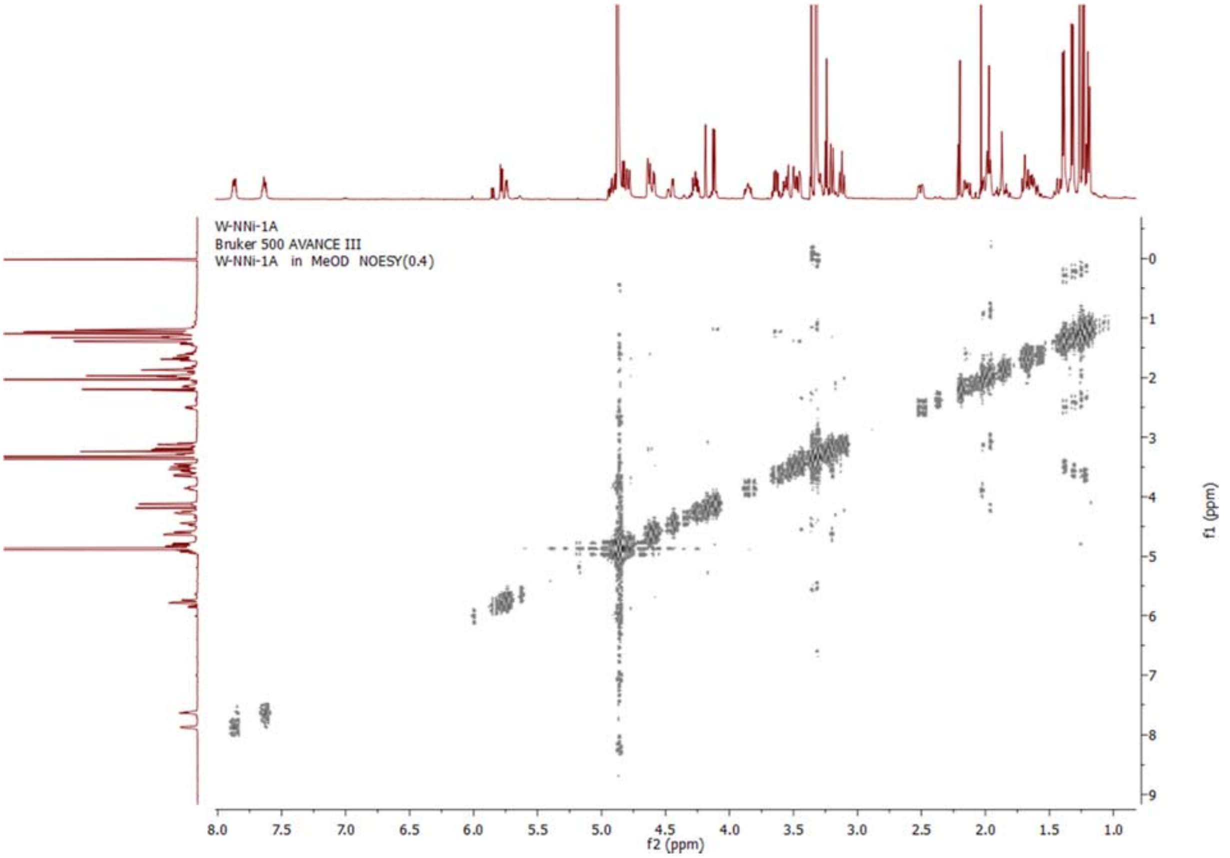

Figure S 15. NOESY spectrum in MeOD for compound **2**.

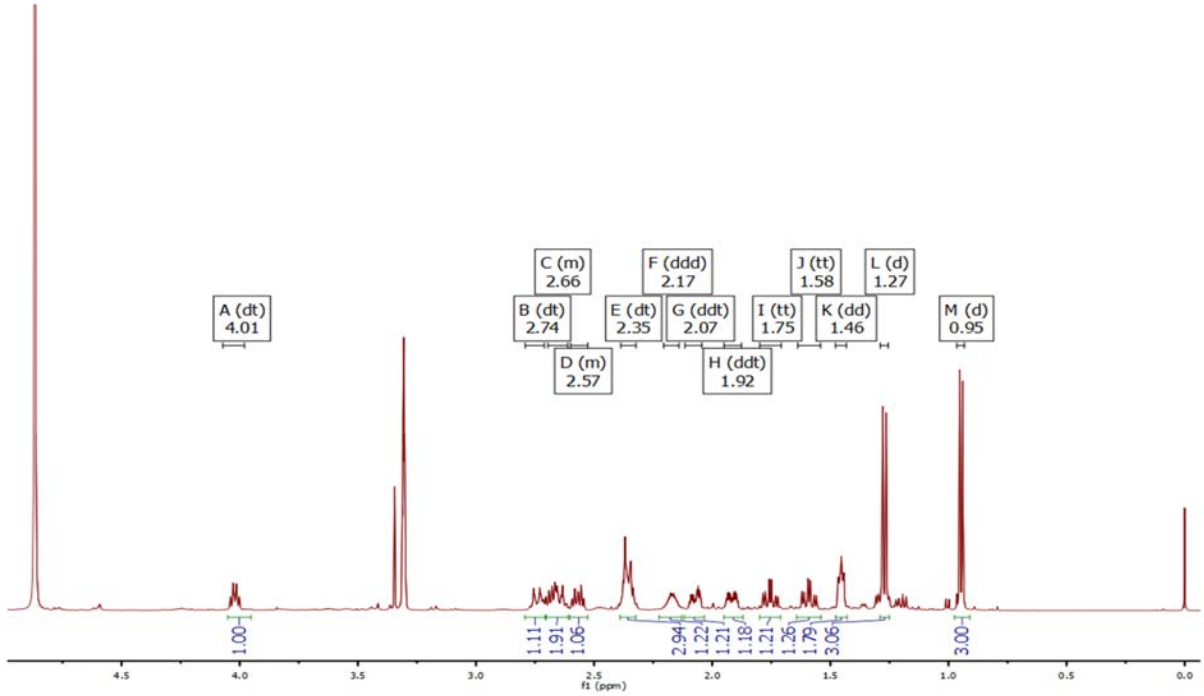

Figure S 16. ^{1}H NMR spectrum in MeOD for compound **3**.

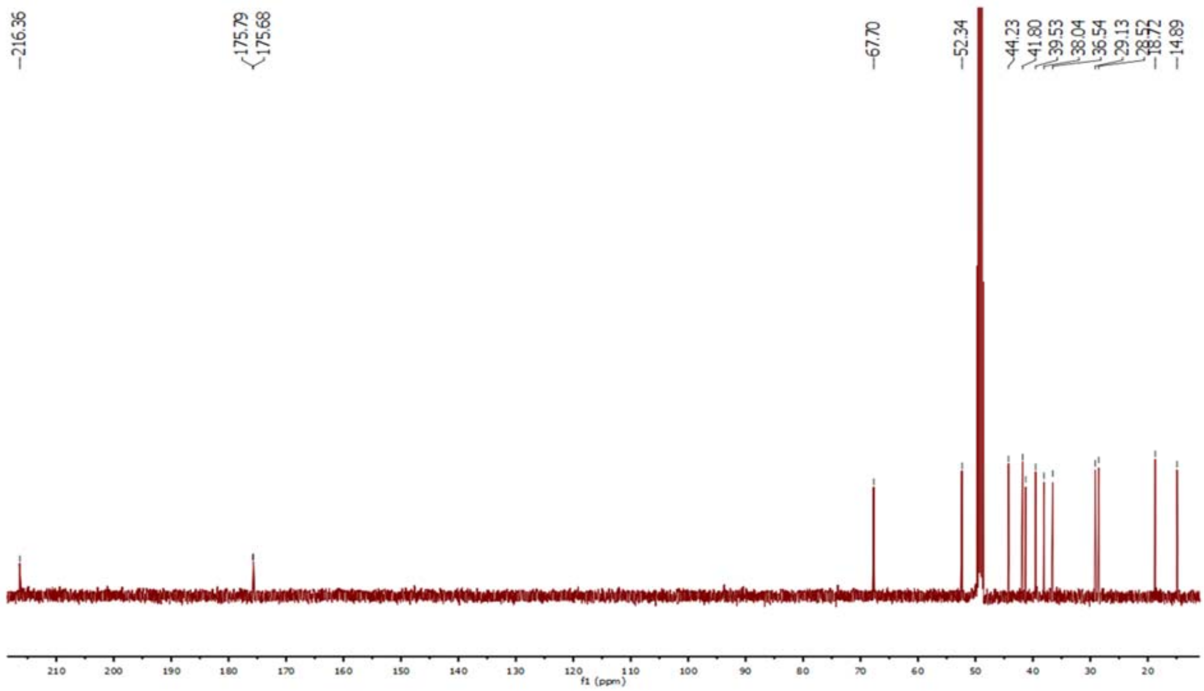

Figure S 17. ^{13}C NMR spectrum in MeOD for compound **3.**

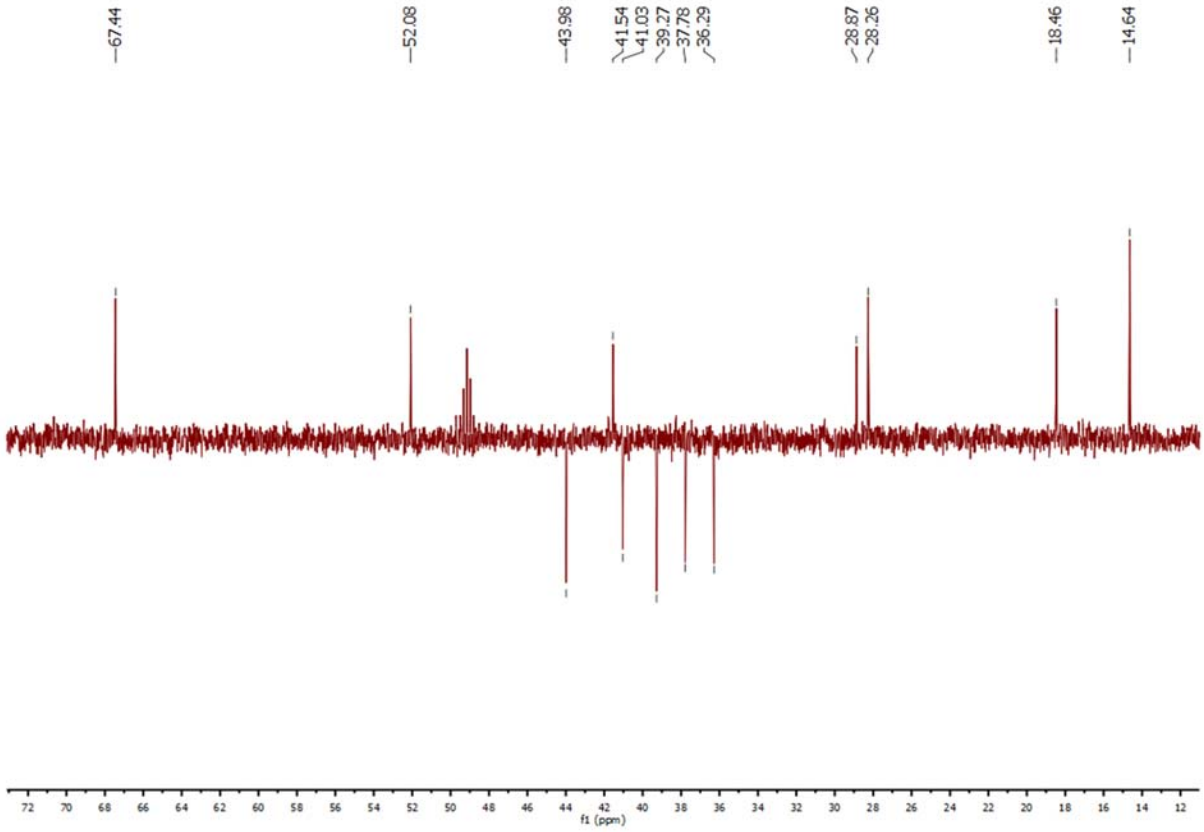

Figure S 18. DEPT-135 spectrum in MeOD for compound **3**.

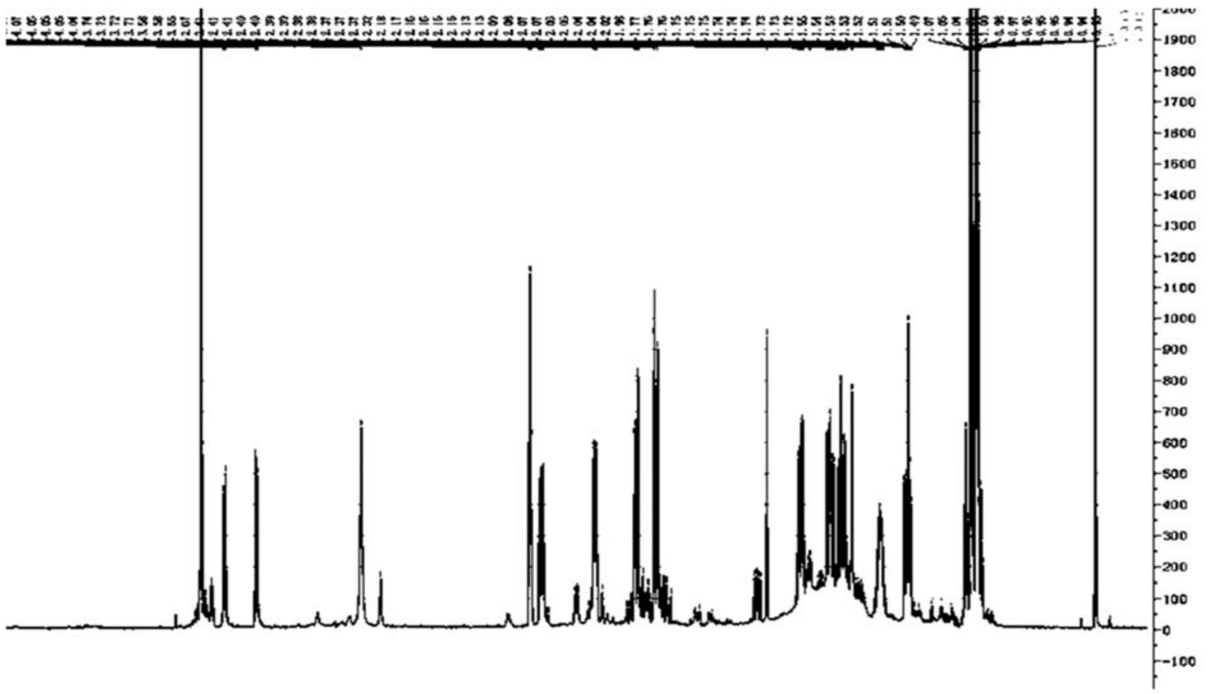

Figure S 19. ^{1}H NMR spectrum in $CDCl_3$ for compound **4**.

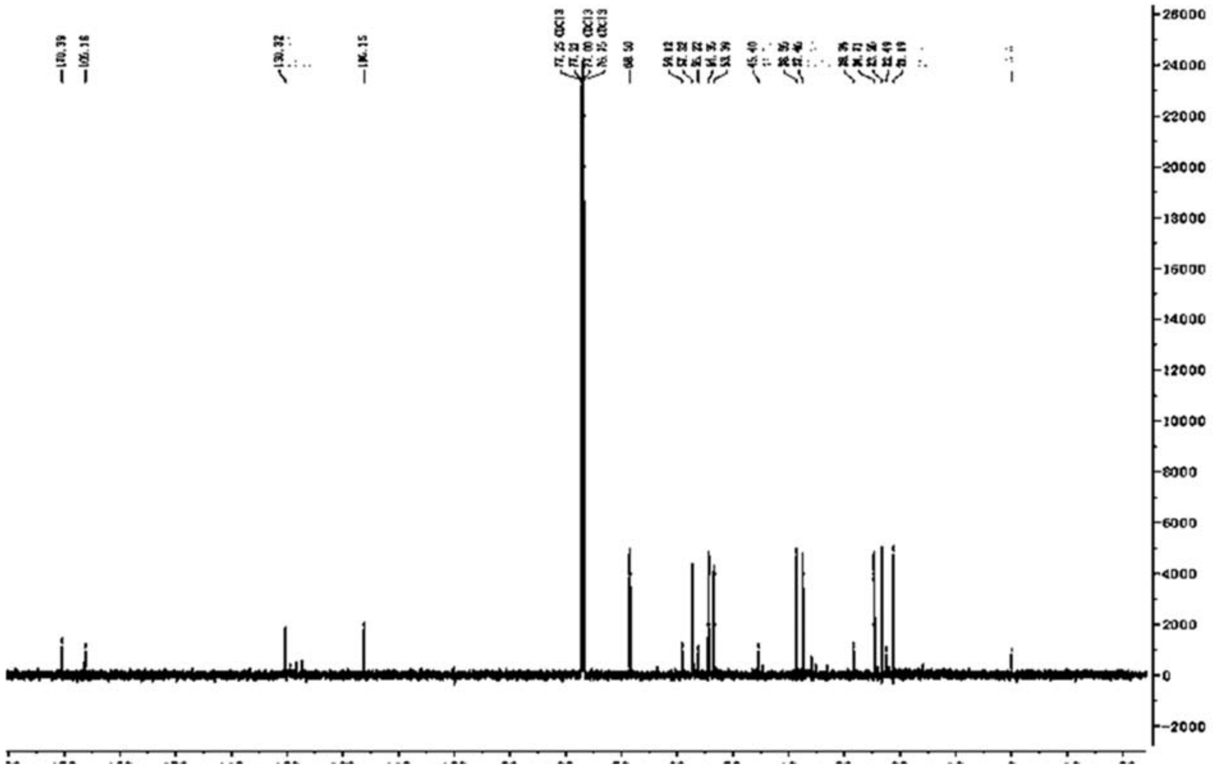

Figure S 20. ^{13}C NMR spectrum in $CDCl_3$ for compound **4**.

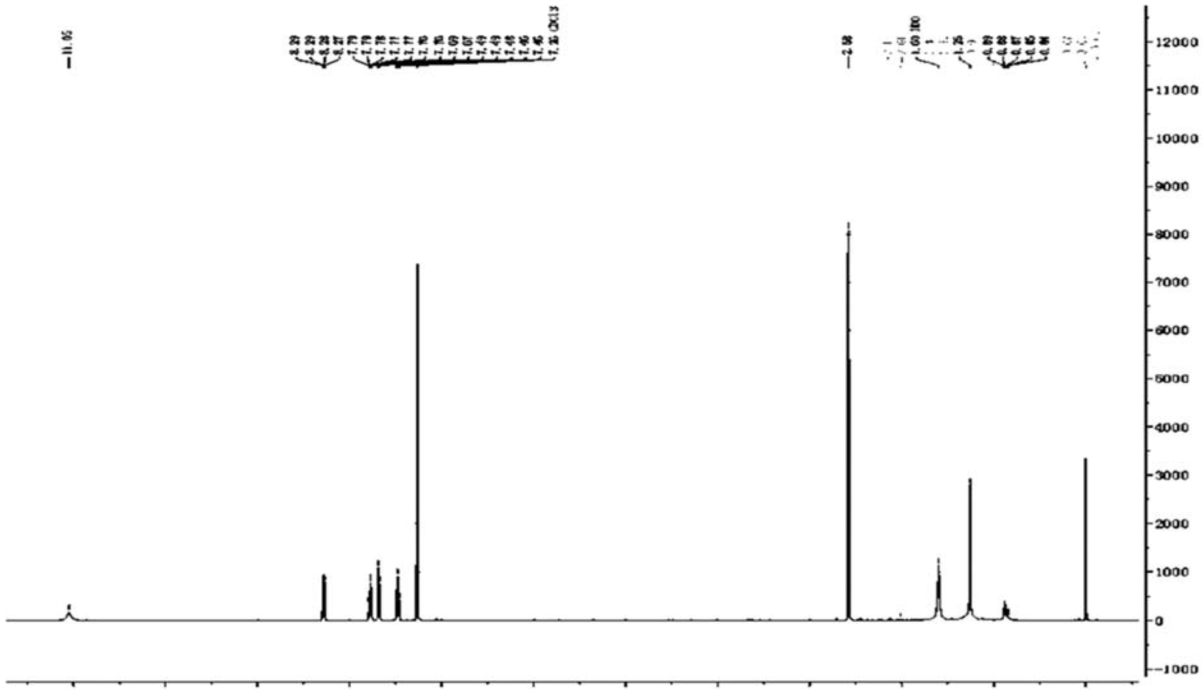

Figure S 21. ^{1}H NMR spectrum in $CDCl_3$ for compound **5**.

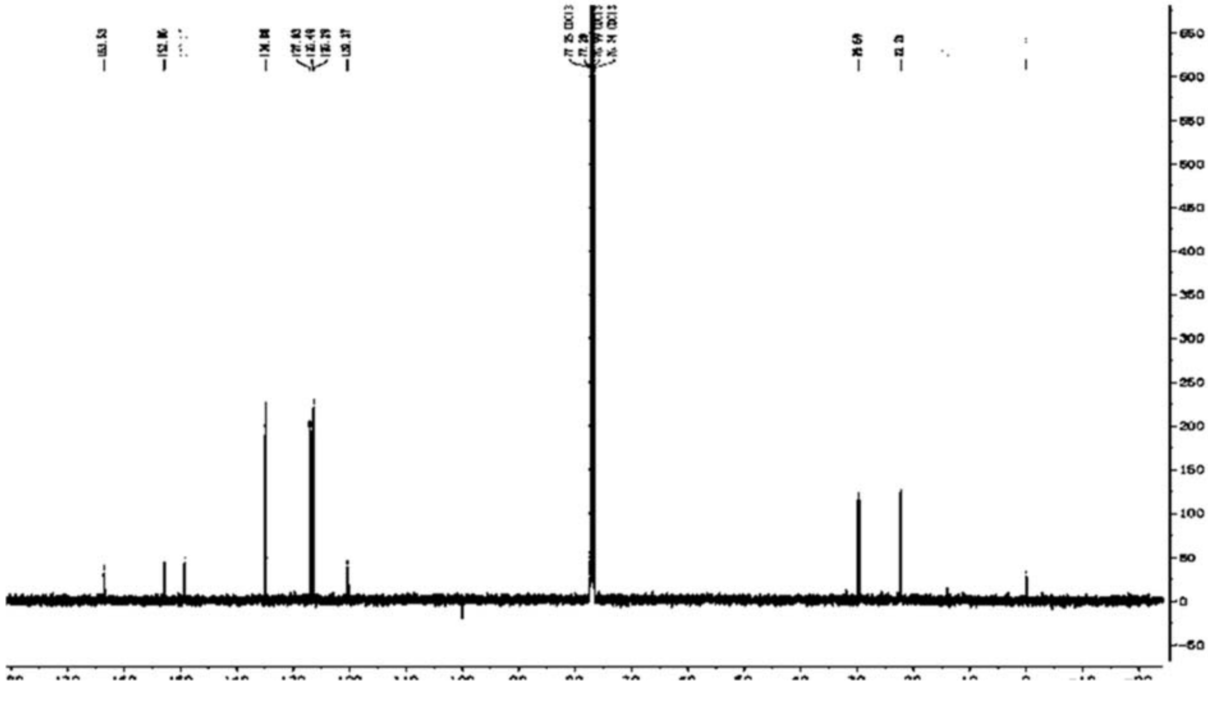

Figure S 22. ^{13}C NMR spectrum in $CDCl_3$ for compound **5**.

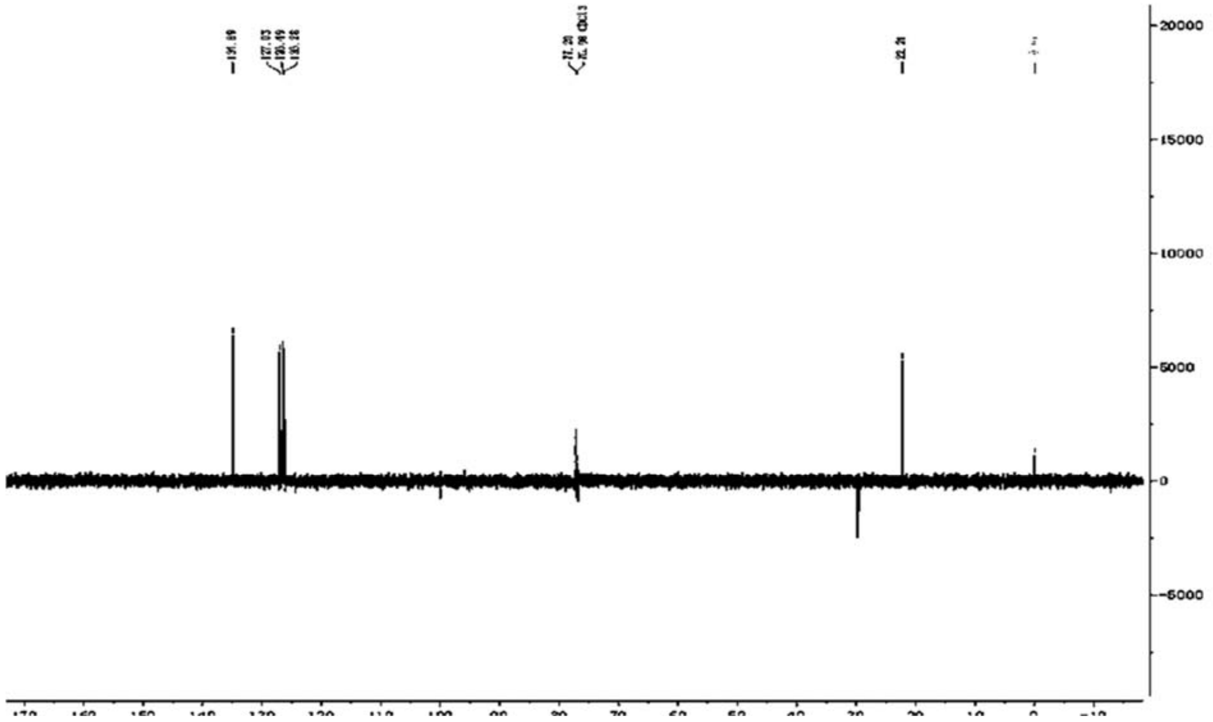

Figure S 23. DEPT spectrum in $CDCl_3$ for compound **5**.

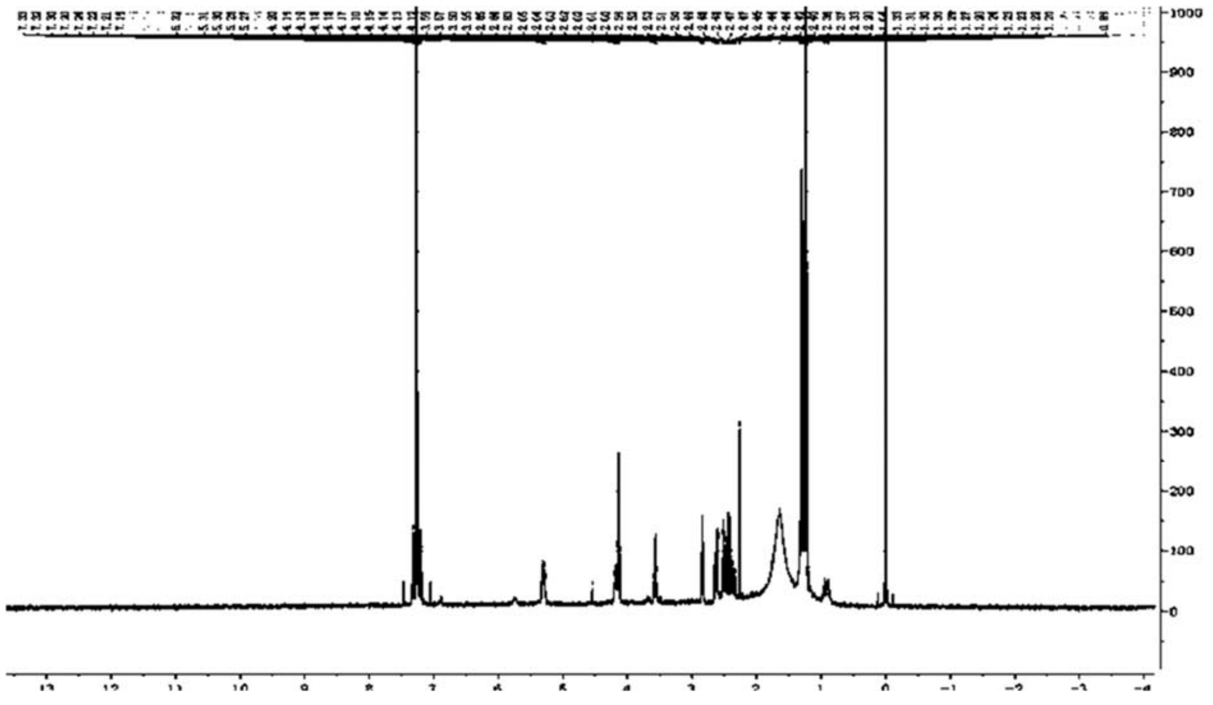

Figure S 24. ^{1}H NMR spectrum in $CDCl_3$ for compound **6**.

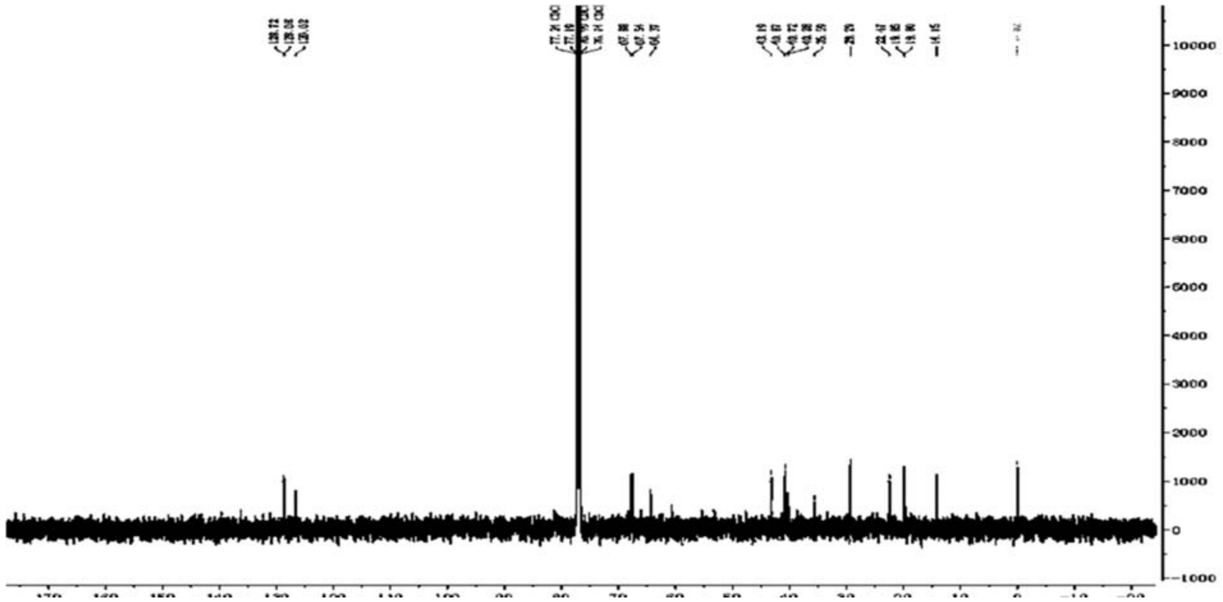

Figure S 25. ^{1}H NMR spectrum in $CDCl_3$ for compound **6**.

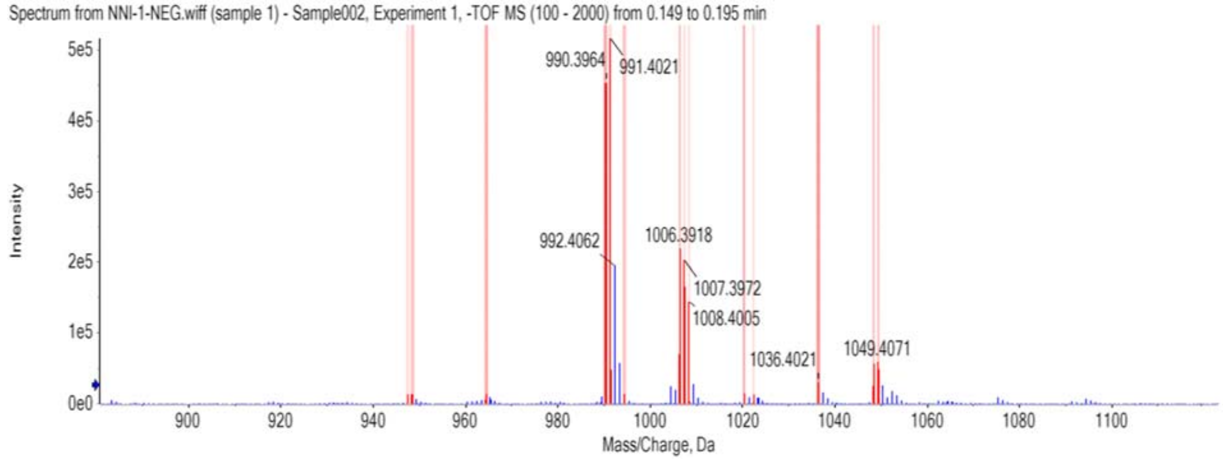

Figure S 26. HR-TOF-MS spectrum in negative mode for compound **1.**

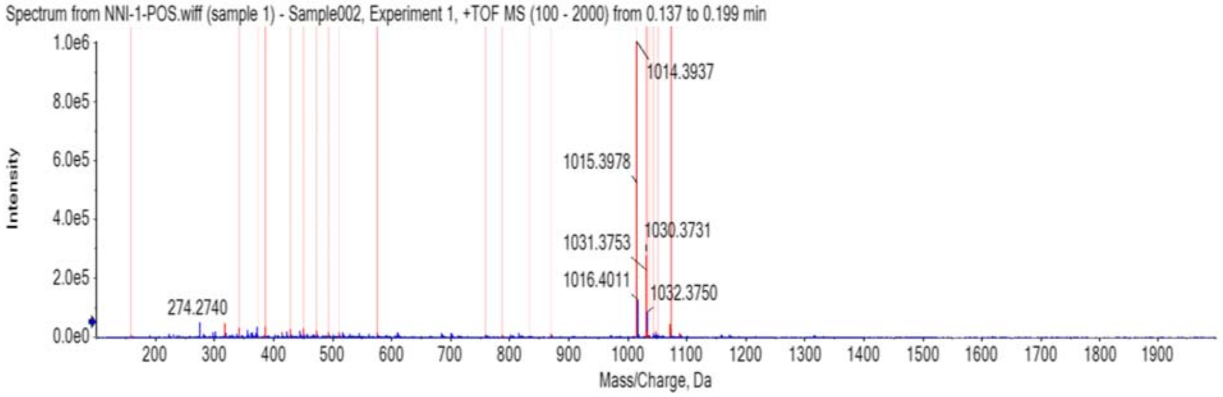

Figure S 27. HR-TOF-MS spectrum in positive mode for compound **1.**

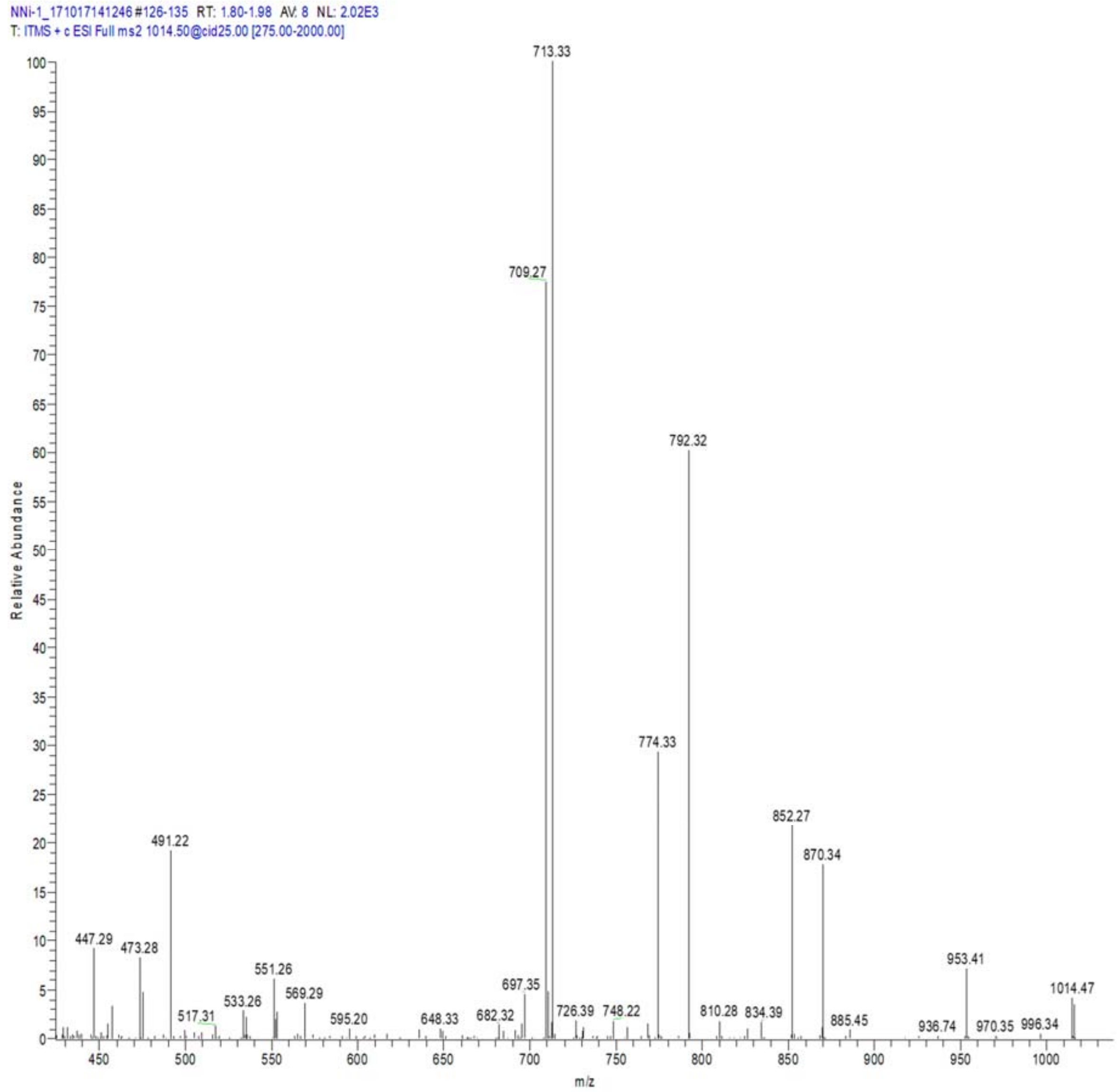

Figure S 28. MS^n Spectrum for compound **1**.

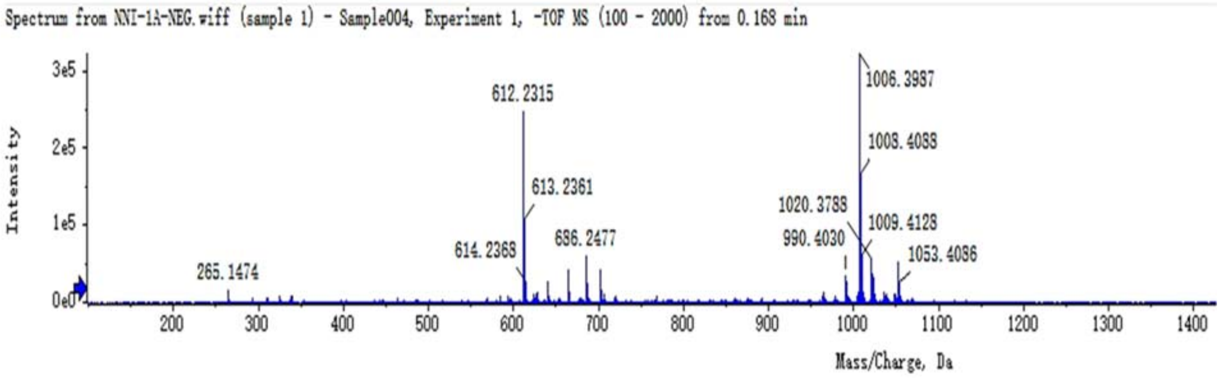

Figure S 29. HR-TOF-MS spectrum in negative mode for compound **2.**

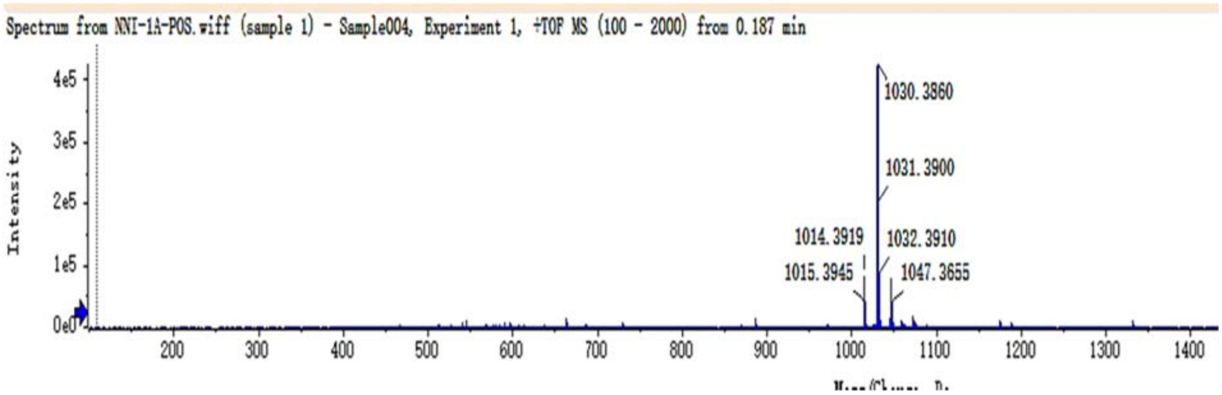

Figure S 30. HR-TOF-MS spectrum in negative mode for compound **2.**

Table S 1. Biological activities of compounds (**1**-**5**) (MIC values are given in µg/mL)

Compounds	MRSA	*Pseudomonas aeruginosa*	*Klebsiella pneumoniae*	*Escherichia coli*	*Bacillus subtilis*
	MIC	MIC	MIC	MIC	MIC
**1**	16	16	16	16	8-16
**2**	16	16	16	16	8-16
**3**	16-32	16-32	16-32	-	-
**4**	32	16	16	16	16
**5**	32	16	16	16	16
Tetracycline	16	32	32	16	8

**Strain**	**16S rDNA gene sequence**
Streptomyces pratensis **NA-ZhouS1**	AGGGCGGGCGTGCTTACCATGCAGTCGAACGATGAAGCCTTTCGG<br>GGTGGATTAGTGGCGAACGGGTGAGTAACACGTGGGCAATCTGCC<br>CTTCACTCTGGGACAAGCCCTGGAAACGGGGTCTAATACCGGATA<br>ACACTCTGTCCCGCATGGGACGGGGTTAAAAGCTCCGGCGGTGAA<br>GGATGAGCCCGCGGCCTATCAGCTTGTTGGTGGGGTAATGGCCTAC<br>CAAGGCGACGACGGGTAGCCGGCCTGAGAGGGCGACCGGCCACA<br>CTGGGACTGAGACACGGCCCAGACTCCTACGGGAGGCAGCAGTGG<br>GGAATATTGCACAATGGGCGAAAGCCTGATGCAGCGACGCCGCGT<br>GAGGGATGACGGCCTTCGGGTTGTAAACCTCTTTCAGCAGGGAAG<br>AAGCGAAAGTGACGGTACCTGCAGAAGAAGCGCCGGCTAACTAC<br>GTGCCAGCAGCCGCGGTAATACGTAGGGCGCAAGCGTTGTCCGGA<br>ATTATTGGGCGTAAAGAGCTCGTAGGCGGCTTGTCACGTCGGATGT<br>GAAAGCCCGGGGCTTAACCCCGGGTCTGCATTCGATACGGGCTAG<br>CTAGAGTGTGGTAGGGGAGATCGGAATTCCTGGTGTAGCGGTGAA<br>ATGCGCAGATATCAGGAGGAACACCGGTGGCGAAGGCGGATCTCT<br>GGGCCATTACTGACGCTGAGGAGCGAAAGCGTGGGGAGCGAACA<br>GGATTAGATACCCTGGTAGTCCACGCCGTAAACGTTGGGAACTAG<br>GTGTTGGCGACATTCCACGTCGTCGGTGCCGCAGCTAACGCATTAA<br>GTTCCCCGCCTGGGGAGTACGGCCGCAAGGCTAAAACTCAAAGGA<br>ATTGACGGGGGCCCGCACAAGCAGCGGAGCATGTGGCTTAATTCG<br>ACGCAACGCGAAGAACCTTACCAAGGCTTGACATATACCGGAAAG<br>CATCAGAGATGGTGCCCCCCTTGTGGTCGGTATACAGGTGGTGCAT<br>GGCTGTCGTCAGCTCGTGTCGTGAGATGTTGGGTTAAGTCCCGCAA<br>CGAGCGCAACCCTTGTTCTGTGTTGCCAGCATGCCCTTCGGGGTGA<br>TGGGGACTCACAGGAGACTGCCGGGGTCAACTCGGAGGAAGGTGG<br>GGACGACGTCAAGTCATCATGCCCCTTATGTCTTGGGCTGCACACG<br>TGCTACAATGGCCGGTACAATGAGCTGCGATGCCGCGAGGCGGAG<br>CGAATCTCAAAAAGCCGGTCTCAGTTCGGATTGGGGTCTGCAACTC<br>GACCCCATGAAGTCGGAGTTGCTAGTAATCGCAGATCAGCATTGCT<br>GCGGTGAATACGTTCCCGGGCCTTGTACACACCGCCCGTCACGTCA<br>CGAAAGTCGGTAACACCCGAAGCCGGTGGCCCAACCCCTTGTGGG<br>AGGAGCTTCGAAGGTGTACGGCAAGTTTCCCTT

Figure S 31. 16S ribosomal DNA gene, full sequence of Streptomyces sp. NA-ZhouS1

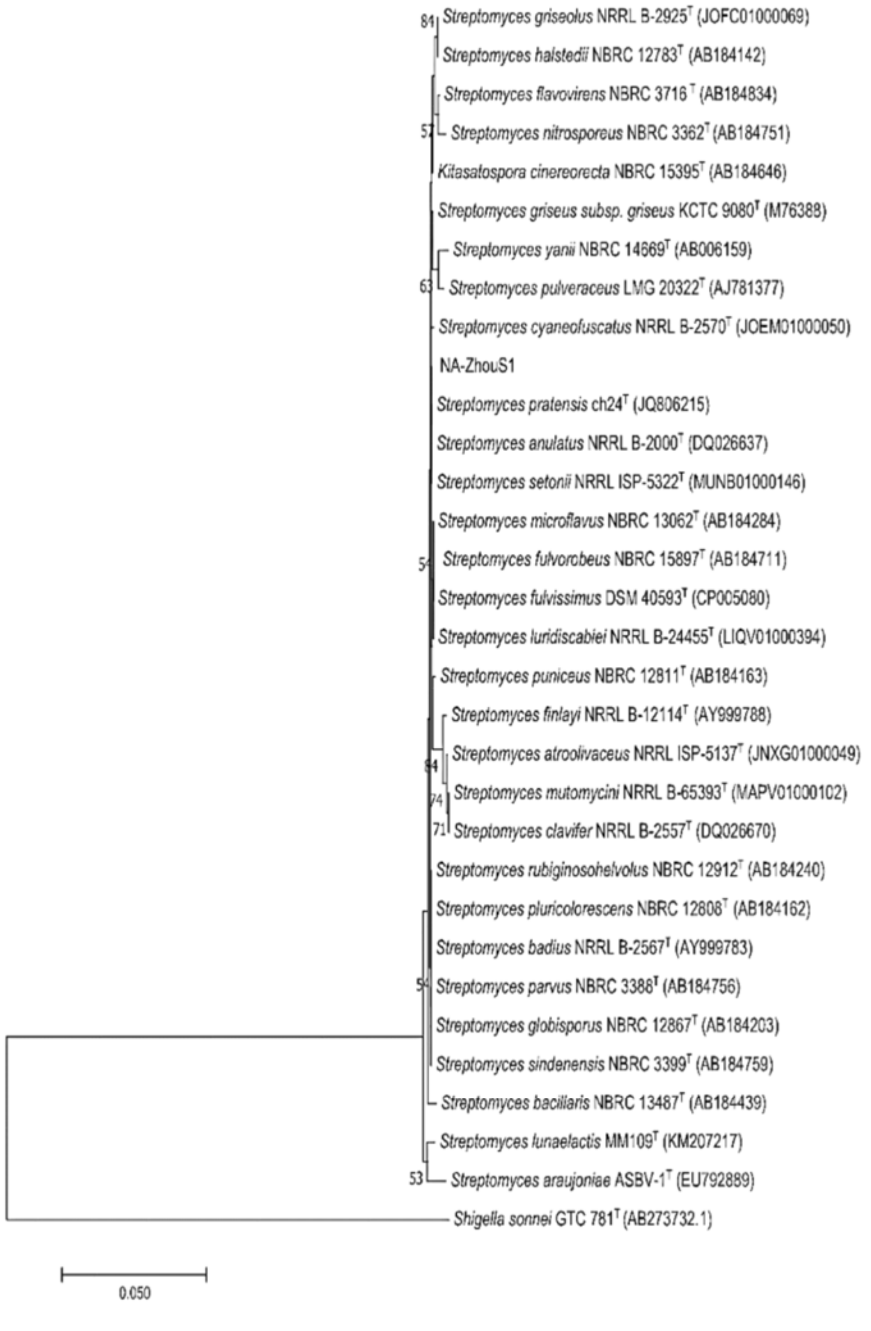

Figure S 32. Neighbor-joining phylogenetic tree of strain NA-ZhouS1 based on 16S rDNA sequences generated by Mega version 7. Numbers at branch points indicate levels of bootstrap (expressed as percentages of 1000 replicates), only values exceeding 50% are given. *Shigella sonnei* GTC 781^T (AB273732.1) was used as an outgroup. Bar, 0.050 substitutions per nucleotide position.
